# A conditional inducible *JAK2^V617F^* transgenic mouse model reveals myeloproliferative disease that is reversible upon switching off transgene expression

**DOI:** 10.1371/journal.pone.0221635

**Published:** 2019-10-10

**Authors:** Emilie A. Chapeau, Emeline Mandon, Jason Gill, Vincent Romanet, Nicolas Ebel, Violetta Powajbo, Rita Andraos-Rey, Zhiyan Qian, Miltos Kininis, Sabine Zumstein-Mecker, Moriko Ito, Nancy E. Hynes, Ralph Tiedt, Francesco Hofmann, Leonid Eshkind, Ernesto Bockamp, Bernd Kinzel, Matthias Mueller, Masato Murakami, Fabienne Baffert, Thomas Radimerski

**Affiliations:** 1 Disease Area Oncology, Novartis Institutes for BioMedical Research, Basel, Switzerland; 2 Friedrich Miescher Institute for Biomedical Research, Basel, Switzerland; 3 Institute for Translational Immunology and Research Center for Immunotherapy, University Medical Center, Johannes Gutenberg University, Mainz, Germany; 4 Chemical Biology and Therapeutics, Novartis Institutes for BioMedical Research, Basel, Switzerland; National Cancer Institute, UNITED STATES

## Abstract

Aberrant activation of the JAK/STAT pathway is thought to be the critical event in the pathogenesis of the chronic myeloproliferative neoplasms, polycythemia vera, essential thrombocythemia and primary myelofibrosis. The most frequent genetic alteration in these pathologies is the activating JAK2^V617F^ mutation, and expression of the mutant gene in mouse models was shown to cause a phenotype resembling the human diseases. Given the body of genetic evidence, it has come as a sobering finding that JAK inhibitor therapy only modestly suppresses the JAK2^V617F^ allele burden, despite showing clear benefits in terms of reducing splenomegaly and constitutional symptoms in patients. To gain a better understanding if JAK2^V617F^ is required for maintenance of myeloproliferative disease once it has evolved, we generated a conditional inducible transgenic JAK2^V617F^ mouse model using the *SCL*-tTA-2S tet-off system. Our model corroborates that expression of JAK2^V617F^ in hematopoietic stem and progenitor cells recapitulates key hallmarks of human myeloproliferative neoplasms, and exhibits gender differences in disease manifestation. The disease was found to be transplantable, and importantly, reversible when transgenic JAK2^V617F^ expression was switched off. Our results indicate that mutant JAK2^V617F^-specific inhibitors should result in profound disease modification by disabling the myeloproliferative clone bearing mutant *JAK2*.

## Introduction

The discovery of the somatic activating *JAK2*^V617F^ mutation in the majority of patients with the chronic myeloproliferative neoplasms (MPNs) polycythemia vera (PV), essential thrombocythemia and primary myelofibrosis marked a breakthrough in the understanding of the underlying molecular mechanism of these diseases [1–4]. Subsequently, gain-of-function mutations in the thrombopoietin receptor *MPL* [5] or *CBL* [6–8], loss-of-function mutations in *SH2B3 [9, 10]*, and most recently frameshift mutations in *CALR* [11, 12] have been discovered in JAK2^V617F^ mutation-negative MPN patients. From a mechanistic standpoint, these mutations have a dysregulated, constitutively activated JAK/STAT pathway in common [13]. Accordingly, transplantation of lethally irradiated mice with murine bone marrow cells transduced with a retrovirus expressing either JAK2^V617F^ or MPL^W515L^ was shown to result in pathological features that closely resemble human PV or myelofibrosis, respectively [14–16]. A phenotype mimicking human essential thrombocythaemia and PV is also obtained upon transgenic expression or knock-in of JAK2^V617F^ in hematopoietic cells of mice [17–22], and it was demonstrated that expression of JAK2^V617F^ in a single hematopoietic stem cell is sufficient to give rise to MPNs [23]. Disease hallmarks observed in the JAK2^V617F^ mouse models include elevation of hemoglobin and hematocrit, leukocytosis, thrombocytosis, megakaryocyte hyperplasia, extramedullary hematopoiesis resulting in splenomegaly, and increased reticulin fibers in the bone marrow of some of the models.

The identification of the JAK2^V617F^ mutation has spurred the discovery and development of JAK inhibitors for the treatment of MPNs [24], and the JAK1/JAK2 inhibitor ruxolitinib received regulatory approval for the treatment of myelofibrosis, and for PV patients who are resistant to or intolerant of hydroxyurea [25–27]. In the clinical setting, ruxolitinib and other JAK inhibitors have shown remarkable activity in terms of suppressing splenomegaly, constitutional symptoms, and aberrant blood counts in MPN patients [24–26, 28, 29]. Similarly, treatment of mouse MPN models with JAK inhibitors, including ruxolitinib, was shown to strongly decrease splenomegaly, and to rapidly normalize red blood cell and neutrophil parameters, consistent with inhibition of constitutive STAT5 phosphorylation in the bone marrow and spleens of treated animals [19, 30–33]. However, it was soon realized that in preclinical models treatment with JAK inhibitors did not substantially affect JAK2^V617F^ mutant allelic burden or eradicated MPN-initiating clones [19, 30, 32, 34]. Similarly, in MPN patients the average reduction of mutant *JAK2* allele burden during treatment with JAK inhibitors was modest, although a subset of patients achieved partial or complete molecular responses [26, 29, 35, 36]. The reason for the limited effect of current JAK inhibitors on the mutant allele burden in MPNs has been subject to debate and is not well understood [37].

To gain more insights into the myeloproliferative disease hallmarks that are dependent on JAK2^V617F^ once the neoplasm has manifested, we generated a conditional inducible transgenic JAK2^V617F^ mouse model. In our model, expression of JAK2^V617F^ is under the control of a tetracycline-responsive promoter element (TRE), and transgene expression was directed to hematopoietic stem and progenitor cells using a tet-off system in which the tetracycline trans-activator (tTA) is under the control of the stem cell leukemia gene (*SCL*) regulatory elements (*SCL*-tTA-2S) [38]. Upon JAK2^V617F^ expression in double-transgenic mice, MPN-like disease closely resembling key aspects of human PV pathology started emerging after a latency period of several months. While the extent of white blood cell (WBC) count elevation as compared to littermate controls was comparable between female and male double-transgenic mice, the female mice displayed a higher elevation of reticulocyte count and more pronounced splenomegaly, whereas a higher platelet count was preferentially seen in male mice. The disease was found to be transplantable, and importantly, was reversible when transgenic JAK2^V617F^ expression was switched off. Taken together, our results corroborate that expression of JAK2^V617F^ in hematopoietic stem and progenitor cells results in a disease phenotype that faithfully mimics the hallmarks of human MPNs and demonstrate that manifested disease is reversible when the mutant *JAK2* allele is inactivated.

## Results

### Generation of a conditional inducible mouse model that allows control of JAK2^V617F^ expression in a doxycycline regulatable manner

A number of preclinical studies have assessed the pathology associated with the expression of JAK2^V617F^ in mouse models. These models included bone marrow transplantation experiments where JAK2^V617F^ expression was driven by retroviral vectors [14, 16, 39, 40], or transgenic mice where JAK2^V617F^ expression was either under the control of a tissue-specific or the endogenous promoter, respectively [19–21]. These studies compellingly corroborated an initiating role for JAK2^V617F^ in the development of myeloproliferative disorders. However, these models did not enable assessing JAK2^V617F^ in MPN maintenance, and blocking JAK2 function was mostly interrogated with systemically acting JAK inhibitors. To overcome these shortcomings, we generated a regulatable murine model, where JAK2^V617F^ transgene expression can be switched on and off in hematopoietic cells (S1A Fig). To this end, a construct was made in which a tetracycline operator drives inducible expression of the human JAK2^V617F^ cDNA and an IRES-EGFP (S1B Fig). This vector was injected into the pronucleus of C57Bl/6 oocytes, and 7 out of 48 transgenic founder lines were genotyped positively and produced progeny. The 7 founders were bred until the F2 generation.

In MPN patients, JAK2^V617F^ is expressed under the endogenous promoter, but uniparental disomy in the MPN clone can select for 2 mutant *JAK2*^*V617F*^ genes and loss of the remaining wild type copy [3, 41]. In order to avoid artificially high expression of the mutant transgene, we determined the relative human *JAK2* copy number in transgenic mice from the 7 founder lines (S1 Table). Founder 29 displayed the lowest copy number of 1.8 on average, suggesting these mice carry two copies of the transgene. We used this mouse line for all future experiments. Primary mouse fibroblasts from this line were cultured *in vitro* and transfected with a CMV-itTA plasmid to evaluate induction of JAK2^V617F^ expression (S2A Fig). A strong increase of transgene expression was found after CMV-itTA transfection, which was blunted when transfected fibroblasts were treated with doxycycline (dox) (S2B Fig), indicating that the expression of JAK2^V617F^ was switchable. By Western blotting, JAK2 protein levels were slightly increased in the itTA transfected samples in absence of dox, and went back to endogenous levels when dox was added (S2C Fig). Although the *GFP* gene was also expressed (S2B Fig), there was no discernible GFP signal using Western blotting (S2C Fig). Sequencing of the transgene from fibroblast genomic DNA revealed construct integrity. When the *JAK2*V617F-IRES-EGFP cDNA construct was transiently co-transfected with itTA into NIH3T3 fibroblasts we readily detected high levels of JAK2 and GFP mRNA expression (S2B Fig), indicating that, unfortunately, in the transgenic context of our model IRES-directed GFP expression may be hampered by the relatively large preceding cDNA [42].

Next, the founder 29 responder line was crossed with the *SCL*-tTA-S2 effector line [38] (S3A and S3B Fig), known to permit reversible on/off switching of transgenes in hematopoietic stem cells and myeloid progenitors [43]. The tTA-2S tetracycline trans-activator under the control of *SCL* regulatory elements (S3A Fig) allows transgene expression in erythrocytes, megakaryocytes, granulocytes and Kit^+^ Lin^-^ hematopoietic cells [38]. In a pilot experiment, we assessed transgene expression and phenotype *in vivo* by following 4 *SCL*-tTA; JAK2^V617F^ double-transgenic and 4 littermate control mice (2 wild type, 2 JAK2^V617F^ littermate controls). Dox containing food was given during gestation and the first weeks after birth to keep the transgene switched off and avoid potential JAK2^V617F^-induced developmental lethality [19]. Mice in the experimental cohort were switched to normal food at 7–9 weeks of age to enable transgene induction. Encouragingly, after an observation period of 138 days off dox, a slight increase in platelet count was discernible in double-transgenic mice as compared with controls (S3C Fig). Mice were sacrificed and spleen weights were also found to be increased in double mutant mice (S3D Fig). Consistent with these phenotypic changes, all double mutant mice exhibited expression of JAK2^V617F^ in spleen (S3E Fig). The transgene copy number was verified again by qPCR in progeny and double mutant mice and remained at around 1 (S3F Fig).

### Conditional inducible expression of JAK2^V617F^ in mice recapitulates key hallmarks of human MPNs

**Fig 1:**
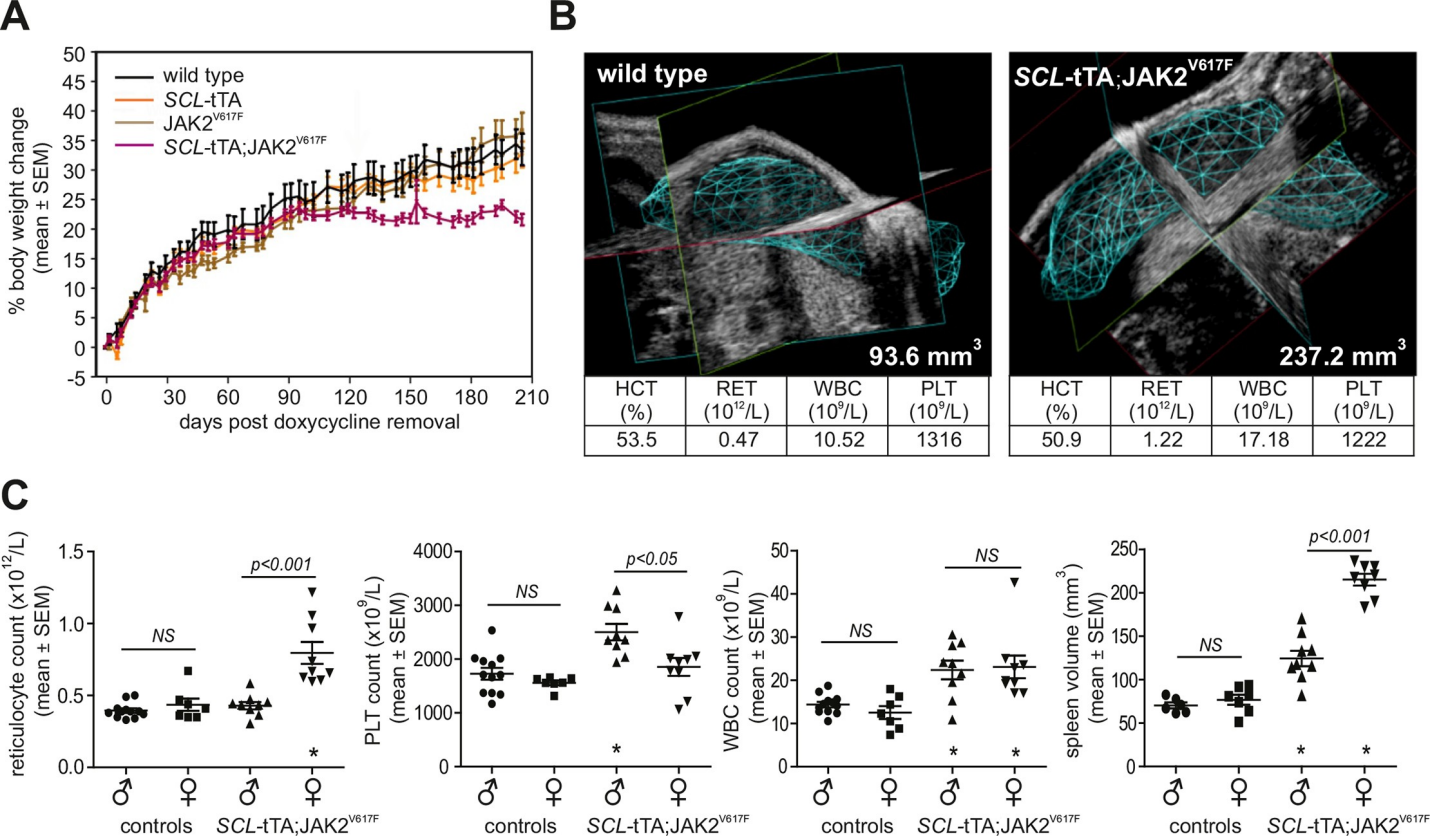
Conditional inducible mouse model that allows control of JAK2^V617F^ expression in a doxycycline regulatable manner. (A) Body weight evolution over time after withdrawal of doxycycline treatment. N = 18–22 animals per group. (B) High-resolution micro-ultrasound imaging of a wild-type and a double-transgenic female mouse at day 207 after doxycycline removal. Spleen volume and blood parameters were measured. (C) Blood counts and spleen volumes at day 207 after doxycycline removal, n = 9–12 mice per group, genders were separated. Unpaired student t-test; *p<0.05 compared to control group. NS: not significant. HCT: hematocrit, RET: reticulocytes, WBC white blood cells, PLT: platelets. Elevated reticulocyte, platelet and WBC counts.

To corroborate these results we crossed further animals from the responder line with *SCL*-tTA-S2 effector mice, and closely followed their development after removing dox in the chow of young adults. After about 6 months post-dox removal body weight gain of *SCL*-tTA;JAK2^V617F^ double-transgenic mice, but not of control littermates, plateaued (Fig 1A). High-resolution micro-ultrasound imaging revealed marked splenomegaly in double-transgenic mice (Fig 1B), along with elevated reticulocyte, platelet and WBC counts (Fig 1C). Interestingly, there was a clear gender difference in the manifestation of MPN phenotypes: While double-transgenic females had more pronounced splenomegaly and higher reticulocyte count, males had a higher platelet count (Fig 1C). Thus, double-transgenic female mice exhibited disease hallmarks of PV, while male mice recapitulated an ET-like phenotype.

**Fig 2:**
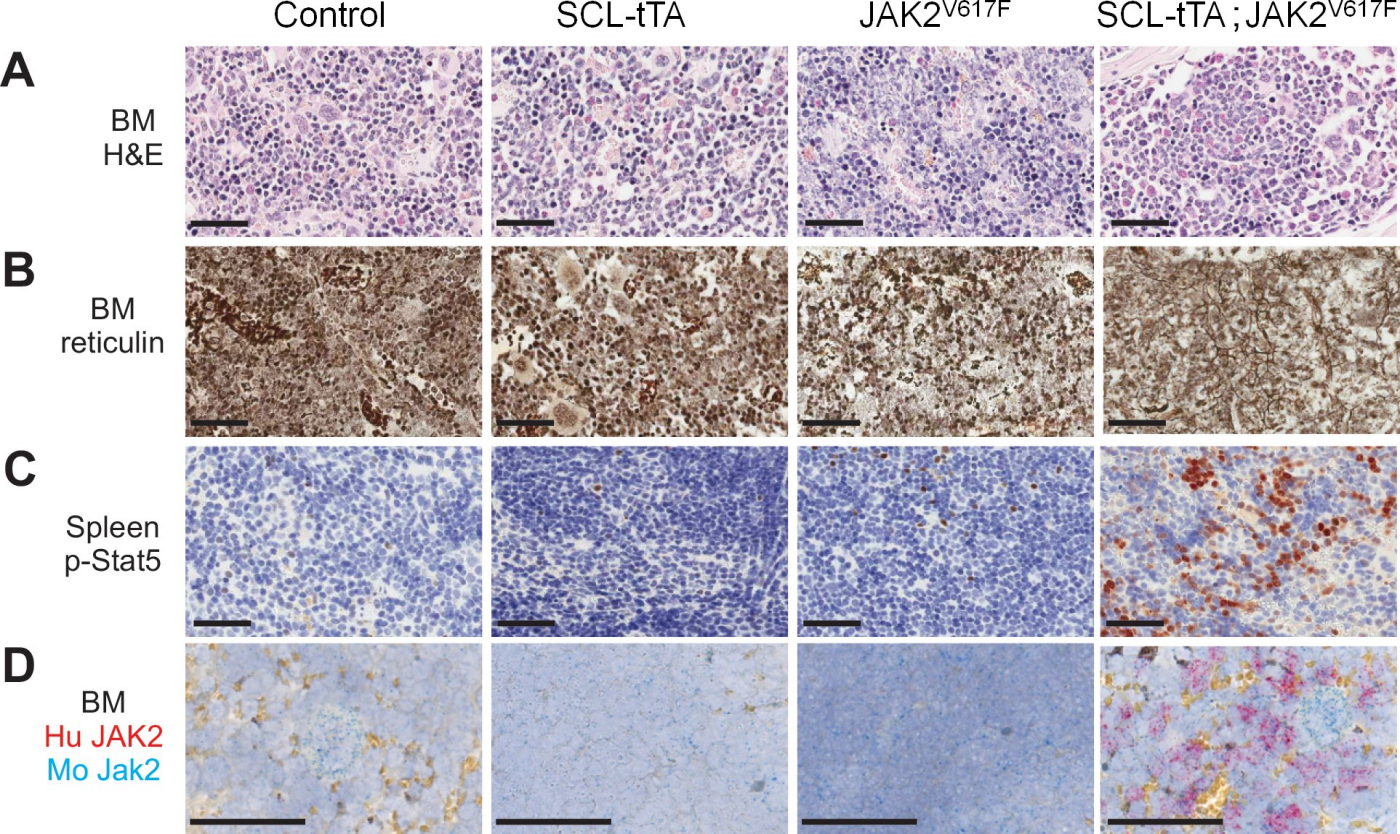
Conditional inducible expression of JAK2^V617F^ in mice recapitulates key hallmarks of human myeloproliferative neoplasm. SCL-tTA;JAK2^V617F^ animals and littermate control mice: wild-type (control), SCL-tTA or JAK2^V617F^, were sacrificed 294 days after doxycycline withdrawal for histopathological assessment of spleen and bone marrow from sternum. (A) H&E staining was performed on bone marrow. Bone marrow of double mutant mice was hyper proliferative. (B) Bone marrow fibrosis was evaluated on the sternum using a silver impregnation staining for reticulin fibers. An increase of reticulin fiber deposits was observed in SCL-tTA;JAK2^V617F^ animals. (C) The phospho-STAT5 marker was assessed in spleen by immunohistochemistry. An increase of phosphorylated STAT5 in the spleen of SCL-tTA;JAK2^V617F^ mice was detected. (D) Expression of JAK2 mRNA was assessed by 2-plex chromogenic in situ hybridization (CISH) (blue for mouse Jak2 and red for human JAK2^V617F^ mRNAs). Bars = 50 um; BM: bone marrow, Hu: human, Mo: mouse.

To further characterize the MPN phenotype, mice were sacrificed after 294 days for histopathological assessment of spleen and bone marrow. Spleens of double-transgenic mice displayed marked erythroid expansion (S4A Fig), and the bone marrow was hyperproliferative (Fig 2A). Furthermore, silver impregnation staining of bone marrow sections showed reticulin fiber deposits in double-transgenic mice but not the littermate controls, which may correspond to a pre-fibrotic stage of human MPNs (Fig 2B and S4B Fig). Immunohistochemistry for phosphorylated STAT5 on spleen sections revealed a strong staining in samples from double-transgenic mice (Fig 2C and S4C Fig), consistent with expression of the JAK2^V617F^ transgene, as detected by chromogenic *in situ* hybridization (CISH) (Fig 2D). Levels of phosphorylated STAT5 were also substantially increased in bone marrow sections of double-transgenic mice, as compared to littermate controls (S4D Fig).

Taken together, these results corroborate that transgenic JAK2^V617F^ expression in the hematopoietic compartment of mice is sufficient to give rise to a phenotype resembling human MPNs.

### The MPN-like disease is reversible upon switching off JAK2^V617F^ expression *in vivo*

Having established that *SCL*-tTA-S2; JAK2^V617F^ double-transgenic mice develop an MPN-like disease several months after induction of JAK2^V617F^ expression raises the key question whether the disease would be reversible upon switching off transgene expression. On one hand, the cooperation of the JAK2^V617F^-bearing MPN clone with its surrounding (JAK2 wild type) stroma sets off a progressive remodeling of the hematopoietic microenvironment in the bone marrow and spleen [44], and it is conceivable that this may lead to gradually reduced dependency on the JAK2^V617F^ initiating event. This might explain the clinical activity observed with current JAK inhibitors in MPN patients, reducing splenomegaly and aberrant blood counts, while having modest effects on JAK2^V617F^ allele burden and on reversing bone marrow fibrosis [19, 30, 32, 34]. On the other hand, it may be possible to not only block the vicious cycle, but also to reverse disease hallmarks, including MPN clonal dominance, by specifically thwarting the MPN clone.

To interrogate these two different possibilities, *SCL*-tTA-S2;JAK2^V617F^ mice that had been fed normal chow for nearly 8 months, as well as their littermate controls, were administered dox-containing food for a period of 72 days. As additional controls, 2 animals of each genotype continued to receive normal chow. Prior to switching mice to dox-containing food, the double-mutant animals exhibited significantly elevated reticulocyte, WBC and platelet counts, as well as increased spleen volume compared to their littermate controls (S5A Fig), showing they had developed an MPN-like disease. Strikingly, after 72 days on dox-containing food all these parameters dropped to the control levels (S5B Fig). The JAK2^V617F^ transcript was undetectable by in situ hybridization on spleen sections of double-transgenic mice that were switched to dox food, while being readily detectable in sections of double-transgenic mice that were kept on normal chow (S5C Fig), demonstrating that JAK2^V617F^ expression can be efficiently switched off again in the transgenic model. The loss of JAK2^V617F^ expression was accompanied by a profound reduction in the number of p-STAT5-positive cells in spleen sections (S5D Fig), and an amelioration of splenic architecture. Importantly, histopathological analysis of bone marrow sections also indicated a marked reduction in reticulin fibers (S5E Fig).

**Fig 3:**
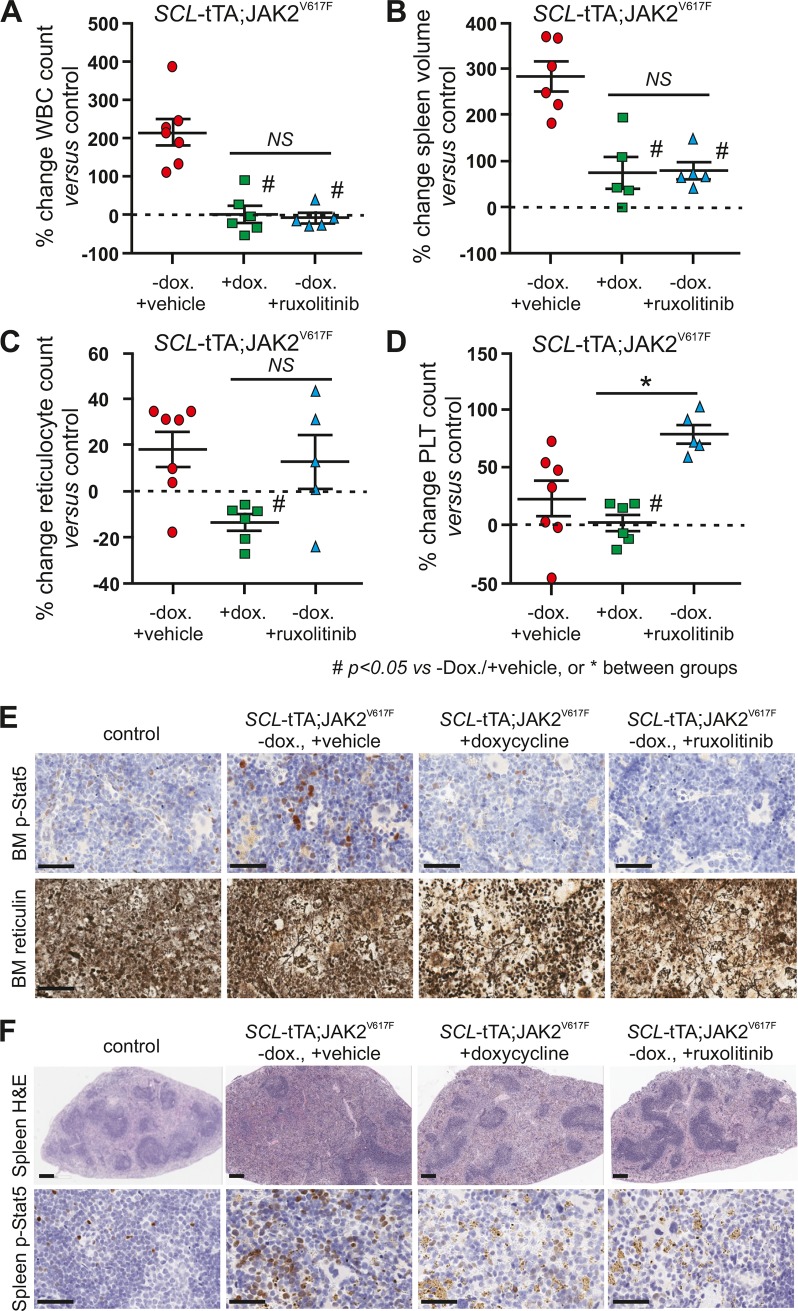
The MPN-like disease of SCL-tTA;JAK2^V617F^ mice is reversible upon switching-off *JAK2*^V617F^ expression or chemically inhibiting JAK2. SCL-tTA/+;JAK2^V617F^/+ mice were aged until they developed MPN-like disease and randomized in 4 groups before being subjected to 26 days of treatment. One group was fed with doxycycline (+dox.) containing chow to turn off JAK2^V617F^ expression, while in the other 2 study cohorts, mice either received ruxolitinib (-dox. +ruxolitinib), or drug vehicle only (-dox. +vehicle). In a fourth arm, wild-type control littermates were also given vehicle (control). Mice were then sacrifized at experimental endpoint and (A) WBC counts and (B) spleen volume, (C) reticulocytes, (D) platelet counts were measured and the % change versus values of control animals was calculated, n = 5–7 mice per group. Mean ± SEM are indicated. Unpaired student t-test #p<0.05 compared to–dox. +vehicle group, or *p<0.05 between groups. NS: not significant, WBC white blood cells, PLT: platelets. (E) The phospho-STAT5 marker was assessed in bone marrow by immunohistochemistry. Bone marrow fibrosis was evaluated on the sternum using a silver impregnation staining. Bars = 50 um. (F) H&E staining was performed on spleens (upper panel, bars = 200 um). The phospho-STAT5 marker was assessed in spleen by immunohistochemistry (lower panel, bars = 50 um).

To confirm and expand on these results, mice from an independent cross were fed normal chow until the *SCL*-tTA-S2; JAK2^V617F^ mice developed MPN-like disease. Then, the double-transgenic mice were randomized into 3 groups: in one arm, mice were switched to dox containing chow to turn off JAK2^V617F^ expression, while in the other 2 study cohorts, mice either received the JAK1/JAK2 inhibitor ruxolitinib at 60 mg/kg twice daily *per os*, or drug vehicle only. In a fourth additional arm, control littermates were also given vehicle. After 26 days of treatment, mice were sacrificed for analysis. WBC counts (Fig 3A) and spleen volumes (Fig 3B) of *SCL*-tTA-S2;JAK2^V617F^ mice were comparably reduced in the dox chow and the ruxolitinib arms. However, reduction of reticulocyte (Fig 3C) and platelet (Fig 3D) counts was superior in the dox chow arm as compared to the ruxolitinib treated group. In fact, platelet count in the latter group was even higher than in the double-transgenic vehicle control group (Fig 3D), an effect which has been observed previously in other mouse MPN models treated with JAK inhibitors [32, 45]. Levels of STAT5 phosphorylation in bone marrow and spleen of double-transgenic mice were markedly reduced in the dox chow and ruxolitinib arms (Fig 3E and 3F). Histopathological analysis of bone marrow sections showed that either turning off JAK2^V617F^ expression or ruxolitinib treatment suppressed marrow hypercellularity and reticulin fibers (Fig 3E and S2 Table). In the spleens of double-transgenic mice, turning off JAK2^V617F^ expression or ruxolitinib treatment both resulted in marked suppression of extramedullary hematopoiesis, along with hemosiderin deposition, which may be the consequence of iron overload [46] once excessive erythroid expansion is blunted (Fig 3F and S2 Table). *In situ* hybridization on spleen and bone marrow sections confirmed that JAK2^V617F^ expression was switched off in double-transgenic mice that were fed dox-containing chow (S6A and S6B Fig). However, in spleens, JAK2^V617F^ expression remained confined to red pulp of ruxolitinib-treated animals (S6A Fig). No change in mouse *Jak2* gene expression was identified in bone marrow within the different treatment arms (S6B Fig). Mouse Jak2 expression was found generally higher in megakaryocytes and fainter in white cells. Ruxolitinib treatment did not induce a compensatory increase of expression of mouse *Jak2* mRNA in megakaryocytes (S6C Fig). Although the JAK1/2 inhibitor suppressed several hallmarks of MPN, including splenomegaly, or reduction of WBC counts, JAK2^V617F^ expressing cells persisted in spleens and bone marrow.

Thus, our data not only show that conditional-inducible JAK2^V617F^ expression is sufficient to give rise to key MPN disease hallmarks, but also that it is necessary to maintain the disease, evidenced by reversibility of the MPN-like phenotype when transgene expression is switched off. Although JAK inhibitor treatment also impressively impacted a number of MPN disease hallmarks in the model, there were differences in several readouts compared to the cohort in which transgene expression had been switched off. Importantly, JAK2^V617F^ expressing cells persisted despite JAK inhibitor treatment, corroborating earlier findings [19], and being seemingly consistent with allele burden data in clinical settings [25].

### Impaired progenitor cell function is reversible upon switching-off JAK2^V617F^ expression

To characterize more precisely the impact of *SCL*-tTA;JAK2^V617F^ phenotypic reversion on hematopoiesis, we analyzed bone marrow cells of mice (S7A Fig) that had been fed normal chow for 275 days. Animals were then distributed in 3 groups: SCL-tTA;JAK2^V617F^ mice were put either off dox, or on dox for 26 days, or treated with ruxolitinib for 26 days, before bone marrow analysis. FACS quantification of Lin^-^ Kit^+^ Sca1^-^ (LKS^-^) progenitors and Lin^-^ Kit^+^ Sca1^+^ progenitors (enriched in hematopoietic stem cells) revealed an increase in the proportion of both types of progenitors when comparing control animals to diseased animals kept off dox all the time, consistent with clinical reports in which JAK2^V617F^ was found to increase amounts of HSC and progenitors [47]. Reversion of JAK2^V617F^ expression for 26 days was not able to revert the increase in these progenitors, whereas ruxolitinib could efficiently revert this effect (S7B Fig). Amounts of common myeloid progenitors (CMP), granulocyte-macrophage progenitors (GMP), and megakaryocyte-erythrocyte progenitors (MEP) were not different in diseased animals expressing the JAK2^V617F^ transgene compared to control animals. However, the reversion of JAK2^V617F^ expression or pharmacological JAK1/2 inhibition for 26 days induced a decrease of MEP in benefit of an increase of GMP cells (S7C Fig).

**Fig 4:**
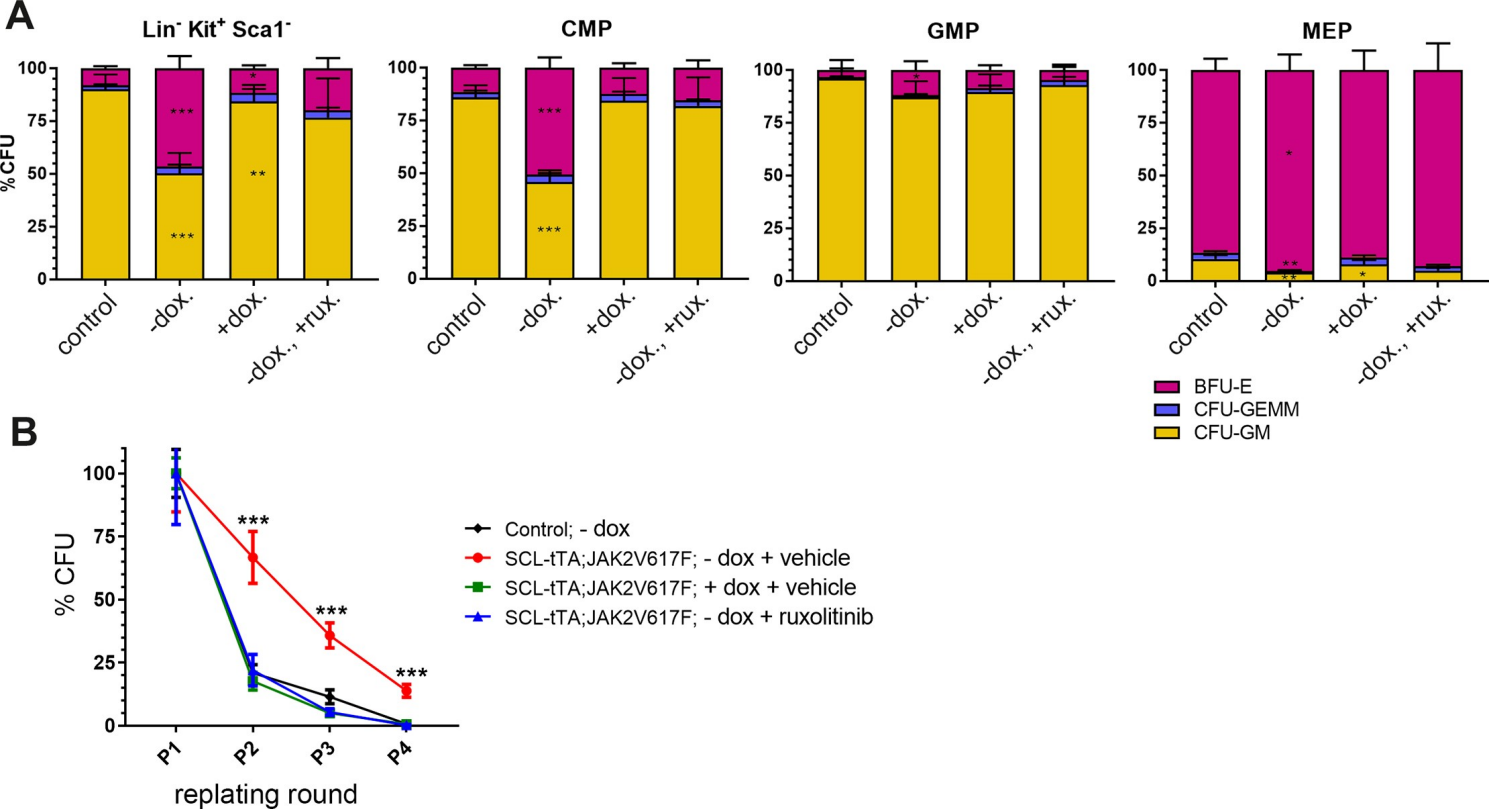
Ex vivo colony-forming assays reveals increased SCL-tTA;JAK2^V617F^ progenitors expansion, reversible upon JAK2 inhibition. SCL-tTA/+; JAK2^V617F^/+ animals were aged more than 8 months without doxycycline and randomized in 4 groups before being subjected to 26 days of treatment. One group was fed with doxycycline containing chow (+dox.) to turn off JAK2^V617F^ expression, while in the other 2 study cohorts, mice either received ruxolitinib (-dox., +rux.), or drug vehicle only (-dox.). In a fourth arm, control littermates were also given vehicle (control). Mice were then sacrifized at experimental endpoint and bone marrow was collected for ex vivo assays. The cells were plated for colony assay on methylcellulose and doxycycline or ruxolitinib treatments were kept as in donor animals. (A) Effects of JAK2 modulations on progenitor populations. Bulk LKS^-^, or CMP, MEP and GMP multipotent progenitors subsets were sorted by FACS from bone marrow and cultured on methylcellulose. CFU-GEMM, CFU-GM and BFU-E colonies were counted after 10 days; n = 14–19 individual assays per group. (B) Serial-replating of isolated LKS^-^ cells was performed; n = 9–12 individual assays per group. Total number of CFU-GEMM, CFU-GM and BFU-E formed colonies on methylcellulose was counted at each round of passage, to assess clonal exhaustion. Mean ± SEM are indicated, P values are from unpaired Student t tests versus control group (*<0.05;**<0.01;***<0.001).

In order to understand the proliferative potential of these progenitors, *ex vivo* colony-forming assays were performed on methylcellulose with LKS^-^, CMP, GMP, or MEP multipotent progenitors isolated from aged mice treated as previously (Fig 4A). Without dox, a markedly increased erythropoietic expansion in SCL-tTA; JAK2^V617F^ was observed in assays performed with various progenitors. This phenomenon was found to be reversible upon switching off JAK2^V617F^ expression or pharmacological JAK1/2 inhibition, since CFU-counts of multipotent myeloid lineage progenitors approached those of littermate controls.

To further investigate whether JAK2^V617F^ may alter repopulating properties, we sorted bone marrow LKS^-^ populations from mice from all groups and performed *in vitro* CFU serial replating assays (Fig 4B). Compared with controls, JAK2^V617F^ expressing colonies showed significantly greater colony formation on replating. Inhibition of JAK2^V617F^ expression or JAK1/2 with ruxolitinib reversed this effect completely. Thus, JAK2^V617F^ reduced clonal exhaustion of multipotent progenitors, but eventually progenitor cell expansion did slow down.

### MPN-like disease is transplantable, and disease manifestation in the host animals can shift compared to the donor phenotype

**Fig 5:**
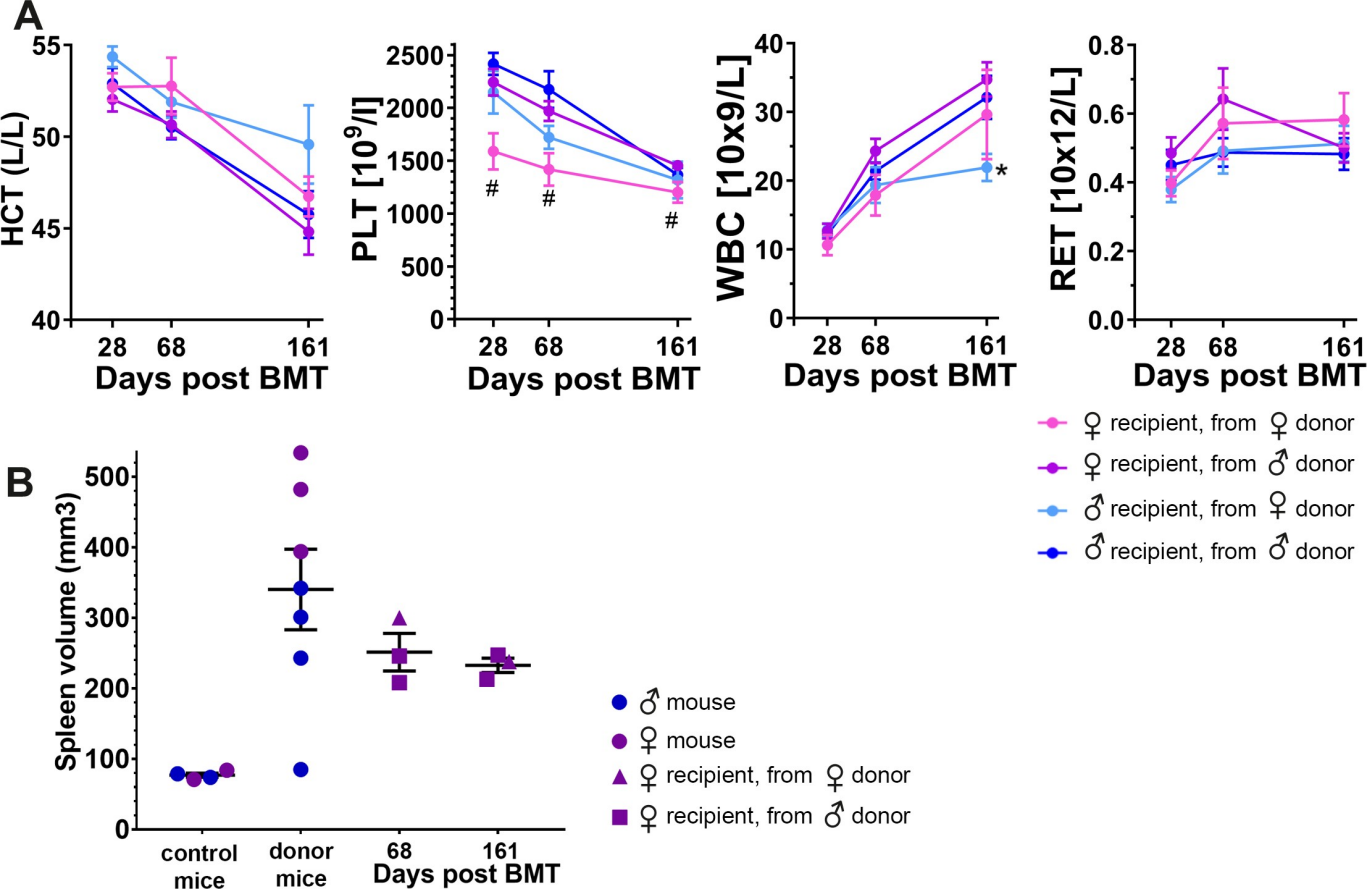
SCL-tTA;JAK2^V617F^-induced MPN-like disease is transplantable. (A) Hemogram results of C57Bl/6 recipient animals at indicated times after bone marrow transplant (BMT) from 13-month-old SCL-tTA;JAK2^V617F^ donor mice. Each curve represents n = 9–12 recipient mice per group, genders are specified. Means ± SEM are used. Unpaired student t-test, *p<0.05 (female donor/male recipient versus male donor/male recipient), #p<0.05 (female donor/female recipient versus male donor/female recipient). HCT: hematocrit, RET: reticulocytes, WBC white blood cells, PLT: platelets. (B) Spleen size of littermate control animals, 13-month-old SCL-tTA/+;JAK2^V617F^/+ donor mice, and BMT recipient mice 68 and 161 days post BMT. Gender are specified. Means ± SEM are used.

Next, we assessed if the JAK2^V617F^ expression-driven MPN-like disease in the transgenic model would be transplantable. To this end, bone marrow cells were harvested from 4 male and 3 female *SCL*-tTA;JAK2^V617F^ mice that had been fed normal chow for nearly 13 months and developed MPN-like disease (S3 Table). Bone marrow cells from each of the respective double transgenic animals were transplanted into six lethally irradiated C57Bl/6J recipient mice (3 female and 3 male recipients). Blood counts were then analyzed 28, 68 and 161 days after transplantation. At the earliest time of analysis platelet count was elevated in most of the transplanted animals (Fig 5A). However, this may be interpreted as a platelet rebound subsequent to irradiation-induced thrombocythemia. Notwithstanding, 40 days later the blood count analysis revealed increases in WBC in the majority of animals. Interestingly, the recipient group without elevated platelet and WBC counts displayed an elevated reticulocyte count (S3 Table). Approximately 9 weeks post-transplantation micro-ultrasound imaging on 3 randomly picked animals showed that spleen volumes were roughly 4 times larger than volumes previously determined in control mice (Fig 5B, S8 Fig, and S3 Table).

By day 161 after transplantation, platelet count had returned to normal levels in the transplanted groups, while WBC count increased in three of four groups when compared to prior readings (Fig 5A). The exception was the recipient group without elevated platelet and WBC counts, and animals in this group continued to have a higher reticulocyte count as compared with the other transplanted groups (Fig 5A). This finding was unexpected, as the animals in this group had been transplanted with bone marrow from the donor mouse that had the highest WBC count, but an unremarkable reticulocyte count (S3 Table). It was also evident that myeloproliferation mainly seemed to involve WBCs, as groups transplanted with bone marrow from donor mice with either increased platelet or reticulocyte counts did not develop thrombocythemia or erythrocytosis. Taken together, these results demonstrate that the JAK2^V617F^ expression-driven MPN-like disease is transplantable, with a bias towards development of leukocytosis, along with splenomegaly.

### Transplanted MPN-like disease is reversible upon switching off JAK2^V617F^ expression

**Fig 6:**
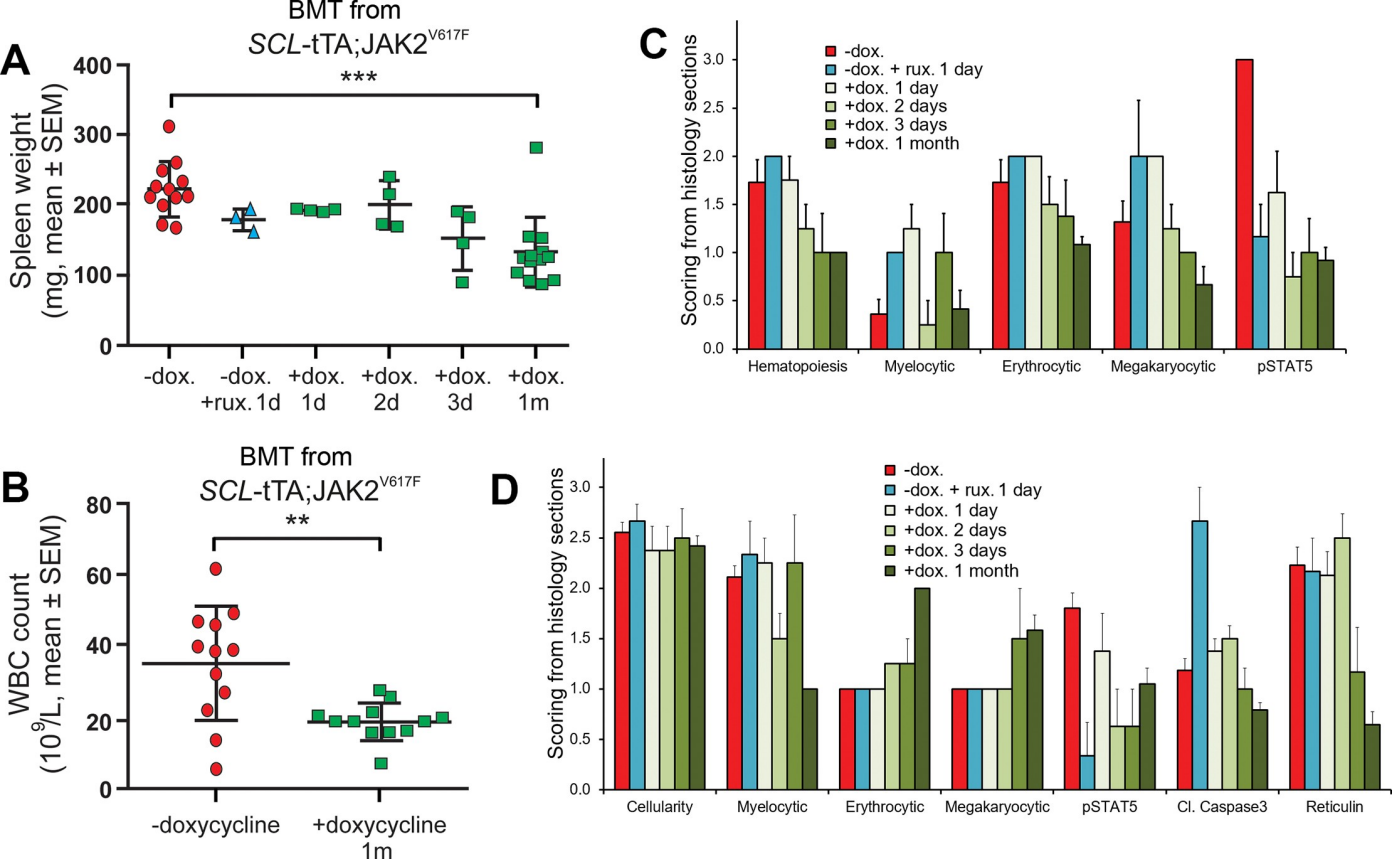
Transplanted MPN-like disease is reversible upon switching-off JAK2^V617F^ expression. Recipient wild-type mice subjected to bone marrow transplant (BMT) from SCL-tTA;JAK2^V617F^ animals were aged more than 5months post BMT without doxycycline before being treated as indicated. Four groups (n = 4–13) were treated for one day (1d), 2 days (2d), 3 days (3d), or a month (1m) respectively with dox (+dox). Another group was treated with 3 doses of ruxolitinib at 12hr interval (-dox. +rux. 1d), and mice were sacrificed 2 hours following the last dose. A control group remained untreated (-dox.). Genders were equally distributed in these groups. (A) Spleen weight was obtained at endpoint. (B) WBC counts were measured at endpoint. Unpaired student t test, **p<0.001 ***p<0.0001. (C-D) Histopathological analysis results of spleen (C) and bone marrow (D) sections. Scoring was used for the quantification of the different hematopietic compartments on H&E sections as well as quantification of specific stainings such as p-STAT5, cleaved Caspase3 and reticulin.

To determine whether the MPN-like disease was dependent on JAK2^V617F^ expression in the transplant setting, transplanted mice were randomized based on hematocrit values into two cohorts of 12–13 mice. They were either continued on normal chow or switched to dox containing chow for one month prior to analyses. The remaining transplanted mice were divided into four cohorts of 4 mice to explore the short-term kinetics of histological and pharmacodynamic marker changes subsequent to modulation of JAK2^V617F^ expression by either diet switching or acute JAK inhibitor treatment. Animals were either administered ruxolitinib at 60 mg/kg (3 consecutive doses, 12 hours apart) or switched to dox containing chow for 1, 2, or 3 days, and given drug vehicle every 12 hours, prior to analyses. In agreement with the notion that JAK2^V617F^ expression is required to initiate and maintain MPN disease, the cohort of transplanted mice switched to dox containing food for one month exhibited reduced spleen weight when compared to the group that continued on normal chow (Fig 6A). Leukocytosis was also significantly suppressed by switching mice to dox containing chow (Fig 6B), while reticulocyte and platelet counts were within normal range (Fig S9A,B). A switch to dox containing chow for 3 days exhibited a trend for spleen weight reduction, demonstrating that the MPN-like disease is impacted rapidly by modulating JAK2^V617F^ expression (Fig 6A).

Histopathological analysis of spleen sections showed reduced extramedullary hematopoiesis in the group switched to dox containing chow for one month (Fig 6C), consistent with decreased spleen weight in this cohort. Analyses of bone marrow histopathology also demonstrated normalization of myeloproliferation, accompanied by expansion of erythroid and megakaryocytic lineages, in this cohort (Fig 6D), which also displayed markedly lower numbers of reticulin fibers in the bone marrow when compared with the cohort on normal chow (S9C Fig). Immunohistochemistry detection of STAT5 phosphorylation was markedly reduced in the bone marrow and spleen of animals in the cohorts switched to dox containing chow (Fig 6C and 6D and S9C Fig), with reduced levels already evident already in the group that was put on dox containing diet for one day. Levels of STAT5 phosphorylation were also substantially lower in the bone marrow and spleen of the cohort treated with ruxolitinib (Fig 6C and 6D and S9C Fig). Cleaved-caspase 3, a marker of apoptotic cell death, exhibited a modest temporal trend with higher levels in the cohort on dox containing chow for less than 3 days and lower levels at later time points. In contrast, acute ruxolitinib treatment increased the cleaved-caspase 3 histology score in the bone marrow by approximately 2-fold, suggesting that the reduction of the phospho-STAT5 signal in the ruxolitinib cohort may be attributable to both JAK2-STAT5 pathway inhibition and induction of cell death, whereas in the cohorts switched to dox containing chow it likely predominantly reflects normalization of JAK2 signaling.

Thus, in the transplant setting continuous expression of JAK2^V617F^ is required to sustain MPN hallmarks and the remodeling of the bone marrow, including prevalence of reticulin fibers, is impacted swiftly upon turning off JAK2^V617F^ expression.

## Discussion

Our present report directly addresses i) disease initiation and ii) disease maintenance using an inducible conditional JAK2^V617F^ allele. By utilizing the *SCL*-tTA-2S tet-off system, we have been able to successfully generate the first conditional inducible mouse model of JAK2^V617F^-driven MPN-like disease. Our SCL-tTA;JAK2V617F mouse model meets the criteria of the Bethesda proposed classification of murine nonlymphoid hematopoietic neoplasms [48] to be defined as a myeloproliferative disease. Indeed, elevated reticulocytes and platelets were observed in peripheral blood (Fig 1, S3 Fig, and S5 Fig). Moreover, histopathology assessment showed increased hematopoiesis and myelopoiesis in spleen and bone marrow, and increased erythrocytic and megakaryocytic expansion in spleens (Fig 2, S4 Fig, and S2 Table). Finally, we demonstrate that this disease is transplantable (Fig 5, S9 Fig, and S3 Table), thus excluding a reactive lesion. Compared to other JAK2^V617F^ mouse models described so far, our model is unique in that it enables assessing reversibility of disease phenotypes upon switching off transgene expression. Double transgenic mice, in which JAK2^V617F^ expression was induced gradually, developed hallmarks of MPN, including elevated blood counts, splenomegaly, bone marrow hyperproliferation and reticulin fiber deposition. Interestingly, female mice exhibited disease hallmarks of PV, characterized by more pronounced splenomegaly and higher reticulocyte count, while male mice displayed a phenotype resembling ET, characterized by an elevated platelet count. To our best knowledge, such gender differences in MPN-like disease manifestation have not been described with other JAK2^V617F^ mouse models. In humans, PV appears to be diagnosed somewhat more frequently in women, manifesting at younger age and exhibiting a higher likelihood to evolve from essential thrombocythaemia as compared to men [49]. The gender differences in MPN-like disease manifestation in our model may be attributable to sex hormones. For instance, estrogen was shown to be responsible for increased hematopoietic stem-cell self-renewal and splenic erythropoiesis in female mice [50], and in a mouse model of JAK2^V617F^-driven MPN-like disease, tamoxifen treatment counteracted the survival advantage of mutant hematopoietic stem and progenitor cells, thereby suppressing disease hallmarks [51]. Our model should allow to genetically test modification of the JAK2^V617F^-induced MPN phenotype by hormone receptors and other genes.

Consistent with previous reports [52], the JAK2^V617F^ expression-driven MPN-like disease is transplantable, manifesting itself quite rapidly in younger recipient mice, and arguing that the disease-causing clone is endowed with multi-potency and self-renewal properties. However, our results also show that a shift in MPN phenotype can occur in recipient mice compared to the MPN-like disease that was originally observed in donor mice. MPN-like disease in transplanted recipient mice predominantly manifested as leukocytosis. The reasons for skewing towards this particular MPN phenotype remain to be determined, but may involve factors such as the initial hematopoietic stress related to the transplant procedure, and/or the interaction that transplanted JAK2^V617F^ expressing hematopoietic stem- and progenitor cells establish with their new stroma and hematopoiesis-inducing microenvironment. In the transplant setting, switching off JAK2^V617F^ expression also led to a reversal of the MPN-like phenotype. While acute JAK inhibitor treatment increased apoptosis in the bone marrow, there was a tendency for a gradual decrease in the number of cleaved-caspase 3-positive cells as compared with baseline upon switching off JAK2^V617F^ expression. The latter finding is seemingly consistent with normalization of both JAK/STAT signaling and hematopoiesis, as aberrant JAK2^V617F^ signaling and myeloproliferation have been attributed to increased reactive oxygen species and DNA damage [23, 53], with ensuing damage and death of cells in the marrow microenvironment [54].

Our transgenic mouse model corroborates that JAK2^V617F^ expression is sufficient to initiate MPN-like disease [17–23]. The fact that disease manifests with high penetrance and largely contemporaneously further supports JAK2^V617F^ as the critical driver. It is conceivable that JAK2^V617F^ triggers epigenetic changes in the MPN clone, and additional mutations may be required for, and acquired during pathogenesis. Seemingly consistent with this notion, additional mutations have been found to co-occur with JAK2^V617F^ in the MPN clone, including mutations in *TET2*, *ASXL1*, *DNMT3A*, *EZH2* or *IDH1/2*, which are thought to impact epigenetic regulation and contribute to clonal skewing [55, 56]. Furthermore, the MPN clone might induce a self-perpetuating remodeling of the hematopoiesis inducing micro-environment, and these stromal changes cooperate with the mutant clone in MPN pathogenesis [44, 57]. Therefore, at least some of the initial dependency on JAK2^V617F^ may be lost during MPN pathogenesis, and JAK inhibition may only be able to tackle those aspects of the diseases that continue to rely on constitutive activation of the JAK/STAT pathway. On the other hand, conditional deletion of *JAK2* in the MPN clone of a mouse model of MPL^W515L^-driven myelofibrosis-like disease was shown to lead to substantially improved disease modification as compared with JAK inhibitor therapy, providing compelling genetic evidence for continued dependency of key pathological features on constitutive activation of the JAK/STAT pathway in the MPN clone [30]. Similarly, in our model of *JAK2*^V617F^-driven MPN we observe full reversibility of MPN-like disease upon turning off transgene expression. This observation demonstrates that continuous expression of JAK2^V617F^ is required to sustain disease.

Collectively, our findings also have important therapeutic implications, arguing for the discovery and development of pharmacological agents that specifically target V617F-mutant JAK2, while sparing the wild type protein. Such agents would be expected to profoundly modify disease in mutation-positive MPN patients. In pharmacological terms, our transgenic mouse model of MPN-like disease, when switched to dox containing diet, may mimic the actions of a V617F mutation-specific JAK2 inhibitor in the context of the heterozygous mutant clone, i.e. with one wild type *JAK2* allele still being present: In the transgenic mouse model JAK2^V617F^ expression is switchable in the background of wild type murine *Jak2*. Thus, when transgene expression is turned off, the cell’s Stat signaling relies again entirely on normal Jak regulation. Our model does not enable us to study the consequences of blunting aberrant Stat signaling in a setting of homozygous JAK2^V617F^ mutation. However, compelling transgenic mouse model studies have established that *Jak2* is essential for hematopoietic stem and progenitor cell functions [58, 59], and that conditionally deleting *Jak2* in the MPL^W515L^-mutant MPN clone profoundly modifies myelofibrosis-like disease, including allelic burden, which has not been achieved to this extent with first-generation JAK inhibitors [30]. Therefore, specifically targeting JAK2V617F in the homozygous mutant MPN clone during the early course of disease might not only have the potential to eradicate clonal dominance, but also the disease, and to enable wild type hematopoietic stem cells to resume normal hematopoiesis.

## Materials and methods

### Generation of the construct and reversible JAK2^V617F^ transgenic mice

The human *JAK2*^V617F^ cDNA was inserted into pCe-lox511-CMVmin and an IRES-EGFP was added. The microinjection was done in male C57Bl/6 pronuclei. Injected embryos were implanted into pseudopregnant females. 48 transgenic founders were obtained and presence of transgene was assessed by PCR. The founder line 29, B6-Tg(TetOCMV-JAK2V617F-IRES-EGFP)29Npa, was used for further experiments.

### Experimental animal studies

All animal studies were conducted in accordance with Kantonales Veterinäramt Basel-Stadt and strictly adhered to the Eidgenössisches Tierschutzgesetz and the Eidgenössische Tierschutzverordnung. The procedures were approved by permit number BS-2139 and BS-2287 and issued by the Kantonales Veterinäramt Basel-Stadt. All animals had access to food and water ad libitum and were identified with transponders. Mice were housed in a specific pathogen–free facility with a 12-h light/12-h dark cycle. Animals were monitored carefully and regularly in all experiments. Mice were euthanized by carbon dioxide overdose.

### Breeding and generation of experimental mice

SCL-tTA-2S knock-in was previously generated and characterized by Bockamp E et al. and named Tal1^tm1(tTA)Bock^ [38]. B6.129S1-Tal1^tm1(tTA)Bock^ was backcrossed to C57Bl/6 background. The B6-Tg(TetOCMV-JAK2V617F-IRES-EGFP)29Npa and B6-Tal1^tm1(tTA)Bock^ mouse lines were then crossed and double heterozygous animals were generated as experimental animals.

### Genotyping of animals

DNA was extracted from tail clips and was digested with proteinase K. SCL-tTA-S2 was detected by PCR (annealing temperature of 58C) with the following primers 5'-ccctgctcgatgccctggc-3' and 5'-aggaaggcaggttcggctcc-3'. An amplicon of 420bp was generated in presence of the knock-in allele. Human JAK2 was genotyped by PCR (annealing temperature of 58C) with the following primers 5'-ACTAAATGCTGTCCCCCAAAG-3' and 5'-CCCATGCCAACTGTTTAGC-3'. An amplicon of 480bp was generated in presence of the transgene.

### Ultrasound imaging of the spleen in vivo and measurement of spleen dimensions

Mice spleens were imaged by ultrasound using a Vevo2100^®^ High‐Resolution Imaging System (VisualSonics, Toronto, Canada). Briefly, mice were starved 3 hours before imaging and fur was removed from the abdomen of mice using depilatory cream (Nair), then the skin was washed with water to remove excess lotion. Mice were maintained anesthetized for the duration of the imaging (1.5% isoflurane; 0.8L O2) on the heated imaging platform to preserve body temperature. With a generous amount of warmed ultrasound gel applied to the skin, the mice were restrained with surgical tape and ultrasound images were taken of its abdomen using the MS500D transducer equipped with 3D Motor. The sagittal and transverse planes are defined. More than 300 individual transverse image slices are obtained and then assembled together on the VevoLAB software for a 3D reconstruction.

### Animal treatments

The expression of the transgene was switched off by doxycycline treatment. Mice were given doxycycline food pellet from Provimi Kliba. The food contained doxycycline at a concentration of 200mg/kg. Ruxolitinib was administrated at 60mg/kg in 0.5% hydroxypropyl methyl cellulose (HPMC) orally twice a day in a solution of 10ml/kg. On the bone marrow transplantation models, dosing of transplanted mice started 21 to 28 days following transplant when disease was established by blood count.

### Bone marrow transplantation

Bone marrow cells were isolated from tibias and femurs of 15-17-month-old SCL-tTA / JAK2^V617F^ mice under sterile conditions. The bones were crushed in a mortar with RPMI + 10% FCS. The medium containing bone marrow cells was filtered through a cell strainer (BD Falcon, # 352340). Cells were centrifuged, the supernatant was removed, and red blood cell lysis buffer was added for 10 minutes at room temperature (Stemcells technologies #07800). Then, RPMI + 10% FCS was added and cells were washed and centrifuged again. Cells were resuspend in pure sterile PBS, counted using Sysmex Nucleocounter, and aliquoted for bone marrow transplantation. 8–9 weeks old C57Bl/6 recipient mice were subjected to total body irradiation (Biobeam, GSM Gamma-Service Medical GmbH) on the day of cells transplantation (2 doses of 4,5Gy at 4hrs interval). Immediately following the second irradiation, mice received a transplant of 5x10^6^ bone marrow cells (from aged SCL-tTA / JAK2^V617F^ double mutant donor animals), injected in tail vein i.v. in a total volume of 100ul in HBSS (Gibco^®^, Cat#14175) at pH 7.4. Body weights were monitored daily for 2 weeks post-transplant. Mice were given antibiotics (Bactrim (Roche Pharma AG) 5ml in 250mL drinking water) to prevent infections for 14 days post bone marrow transplant, and DietGel Boost.

### Blood counts

Blood was collected from the tail vein using Microvette 100K3E tubes with EDTA (SARSTEDT, cat#. 20.1278) for blood analysis. Blood parameters (red blood cells, white blood cells, platelets, and reticuocytes counts) were determined on Sysmex blood analyzer.

### Histopathological assessment

Sterna, femurs and spleens were collected, fixed in formalin (10% neutral buffered formalin) for 48 hours at room temperature, EDTA decalcified for 3x24 hours at 37°C for bones, rinsed in PBS, processed for dehydration, cleared and paraffinized. After embedding in paraffin, 3μm sections were prepared. H&E staining was performed on all organs. Bone marrow fibrosis was evaluated on the sternum using a silver impregnation kit for reticulin fibers (Bio-Optica, #04–04080).

### Immunohistochemistry

3um thickness tissue section were stained on a BondRX platform (Leica Biosystems) using Epitope Retrieval 2 (ER2) conditions for 20mn at 100°C for all tested markers. The detection system used was either Refine DAB kit (Leica Biosystems) for p-STAT5 and Cleaved Caspase 3 immunohistochemistry or Refine Red kit (Leica Biosystems) for GFP immunohistochemistry. Slides were hematoxylin counterstained, dehydrated and cover slipped. Primary antibodies used were anti-phospho-STAT5 (clone C11C5, CST, #9359) or cleaved Caspase3 (CST, #9661) or GFP (MBL, #598).

### mRNA in situ hybridization

*In situ* hybridization was performed using either the 1-plex Quantigene View RNA ISH Tissue assay kit (Affymetrix, #QVT0051) or the 2-plex Quantigene View RNA ISH Tissue Assay kit (Affymetrix, #QVT0012). Assays were performed as per the manufacturer’s instructions with stock solutions. Before beginning the hybridization protocol, 5um thickness tissue section were baked at 60°C for 30 min to increase tissue adhesion. The paraffin was removed from the slides with xylene before being boiled in a pretreatment solution (Affymetrix) for 10 min and incubated with Protein Kinase K (Affymetrix) at 40°C for 10 min. Custom probes for *Human JAK2* and *Mouse Jak2* (Affymetrix) were then hybridized to the tissue. Signal amplification was accomplished by hybridizing Type1 and Type2 specific pre-amp oligonucleotides, amp oligonucleotides and labeled oligonucleotides sequentially. Slides were then mounted with an aqueous mounting medium (Dako, Ultra mount). Digital slide image data was generated from the glass slides using the Aperio AT2 slide scanner (Leica Biosystems) for bright field, and the P250 Scanner (3D Histech) for fluorescent acquisition.

### Flow cytometry analysis and sorting

Multicolour analysis for progenitor and stem-cell quantification was performed on a FACS-Aria (BD Biosciences, San Jose, CA). Cells were stained in PBS 2%BSA with CD34–FITC (1:100), CD150–PE (1:100), FcγRIII/II-PercpCy5.5 (1:400), CD135-PECY5 (1:100), CD117-PECy7 (1:200), TER119-APCCy7 (1:500), lineage cocktail-APC (B220/CD19/CD3/CD4/CD8/NK1.1/TER119/CD11b/Gr1), Sca1-biotin (1:500), streptavidin-Brilliant Violet 605 (1:500) and 1 μg ml-1 DAPI. FACS enrichment of LKS, GMP, CMP and MEP was performed on the FACS ARIA and data analyzed using FlowJo software (Tree star, Ashland, OR). Briefly, bone marrow (depleted of red blood cells) was labeled with APC-conjugated mature blood cell lineage-specific antigens CD3, CD4, CD8, Gr-1, Mac-1, B220, CD19 and TER119. Doublets, DAPI positive dead cells, and cells expressing lineage markers were excluded from the analysis. More details in Supporting Materials and methods.

### Hematopoietic cell colony-forming assays

Mouse tibiae were used to extract bone marrow cells and phenotypically defined hematopoietic progenitor-cell populations were obtained by FACS. Myeloid lineage colony-forming assays were performed in SCF, IL-3, IL-6, EPO supplemented methylcellulose-based medium (Methocult M3434, Stem Cell Technologies). Doxycycline (1ug/ml) and ruxolitinib (1μmol/l) were applied to the medium where indicated and replaced every three days. For LKS-, CMP, GMP and MEP methylcellulose cultures, 2000 cells were seeded in duplicate and scored for colony formation at 7–10 days. For serial replating experiments, LKS+ cells were plated at 2,000 cells per well and scored for colony formation after 10–12 days of primary culture. Cells were subsequently counted and resuspended in fresh medium with 2 x 10^5^ cells replated for each of three rounds of serial replating. Serial plating efficiency was scored as percentage of initial CFU per well obtained at 10–12 days culture.

## Supporting information

S1 Fig Generation of a conditional inducible mouse model that allows control of JAK2V617F expression in a regulatable manner. (JPG)

S2 Fig Primary mouse fibroblast culture assay to screen for transgene inducibility. (JPG)

S3 Fig Pilot in vivo screen with SCL-tTA;JAK2V617F double mutant mice. (JPG)

S4 Fig Conditional inducible expression of JAK2^V617F^ in mice recapitulates key hallmarks of human MPNs. (JPG)

S5 Fig Conditional inducible transgenic mouse model demonstrates that MPN-like disease is reversible upon switching-off JAK2^V617F^ expression. (JPG)

S6 Fig Jak2 gene and transgene expressions upon switching-off JAK2^V617F^ expression. (JPG)

S7 Fig Phenotypic reversibility assessment by flow cytometry profiling of bone marrow hematopoietic progenitors. (JPG)

S8 Fig Ultrasound imaging and volume measurements of spleens of transplanted animals. (JPG)

S9 Fig Transplanted MPN-like disease is reversible upon switching-off JAK2^V617F^ expression. (JPG)

S1 Table Human JAK2V617F copy number determination in founders. (JPG)

S2 Table Histopathological analysis of spleen and bone marrow sections demonstrate the reversibility of SCL-tTA;JAK2V617F phenotype. (JPG)

S3 Table SCL-tTA/+;JAK2^V617F^/+ induced MPN-like disease is transplantable and disease manifestation in the host animals can shift compared to the donor phenotype. S1 Methods
(JPG)

S1 Methods (DOC)
