# Supplementary figures and images for: A conditional inducible JAK2V617F transgenic mouse model reveals myeloproliferative disease that is reversible upon switching off transgene expression
Source: PLoS One. 2019 Oct 10;14(10):e0221635. doi: 10.1371/journal.pone.0221635 (PMC6786561; doi:10.1371/journal.pone.0221635)

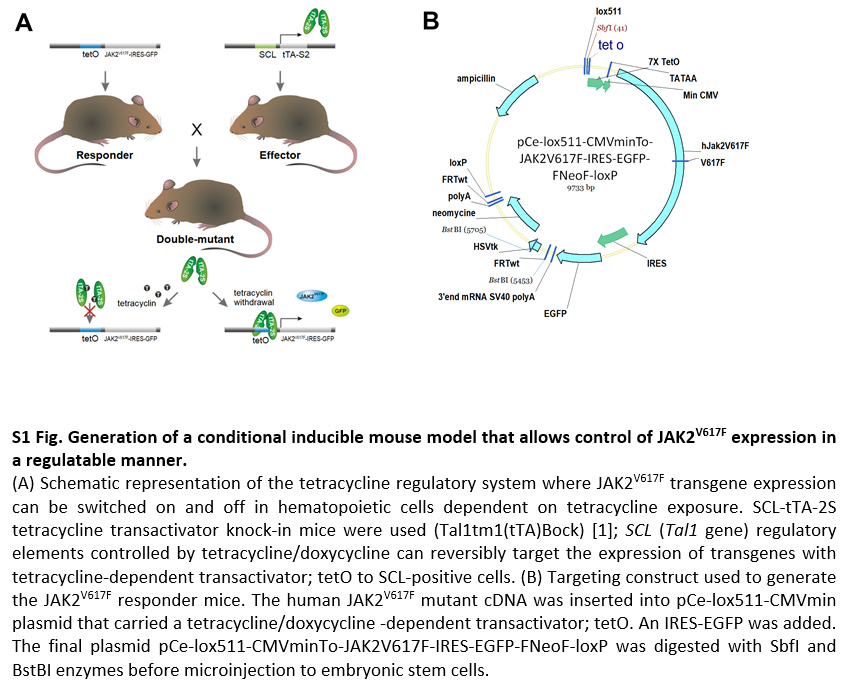

Supplement: S1 Fig — (JPG) [file pone.0221635.s001.jpg]

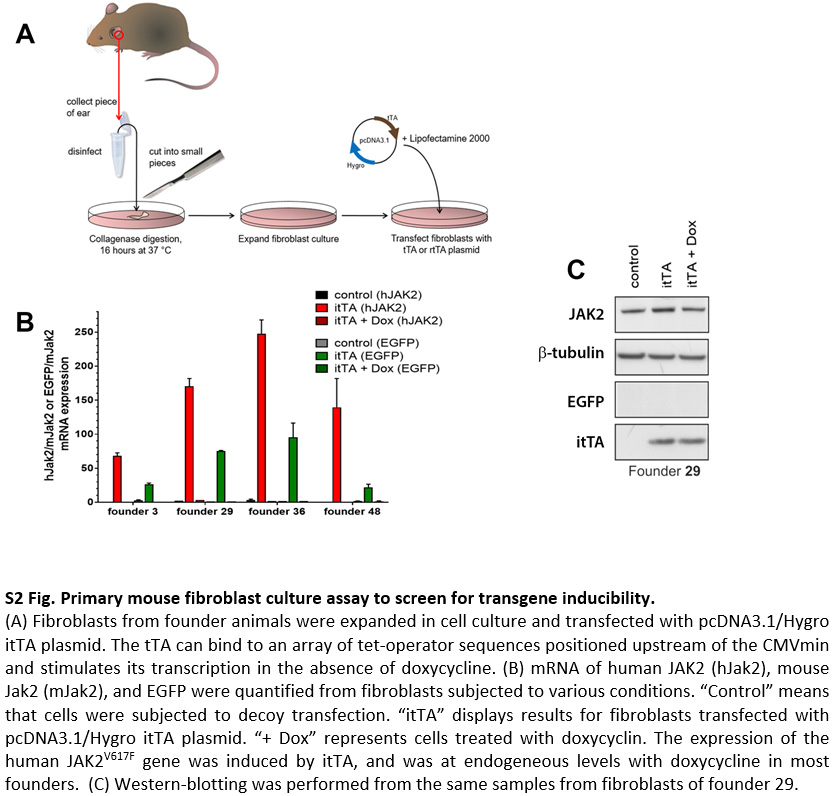

Supplement: S2 Fig — (JPG) [file pone.0221635.s002.jpg]

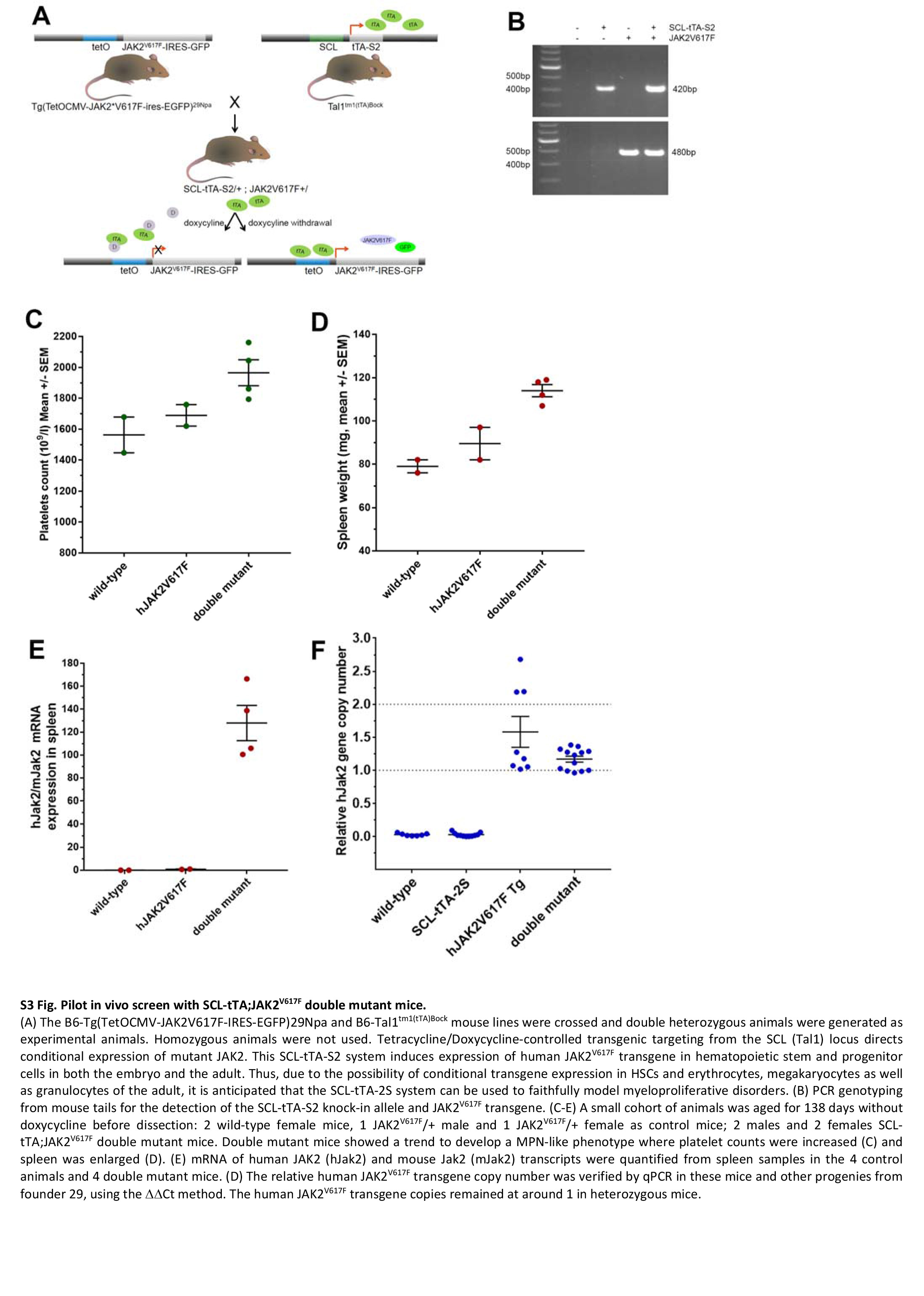

Supplement: S3 Fig — (JPG) [file pone.0221635.s003.jpg]

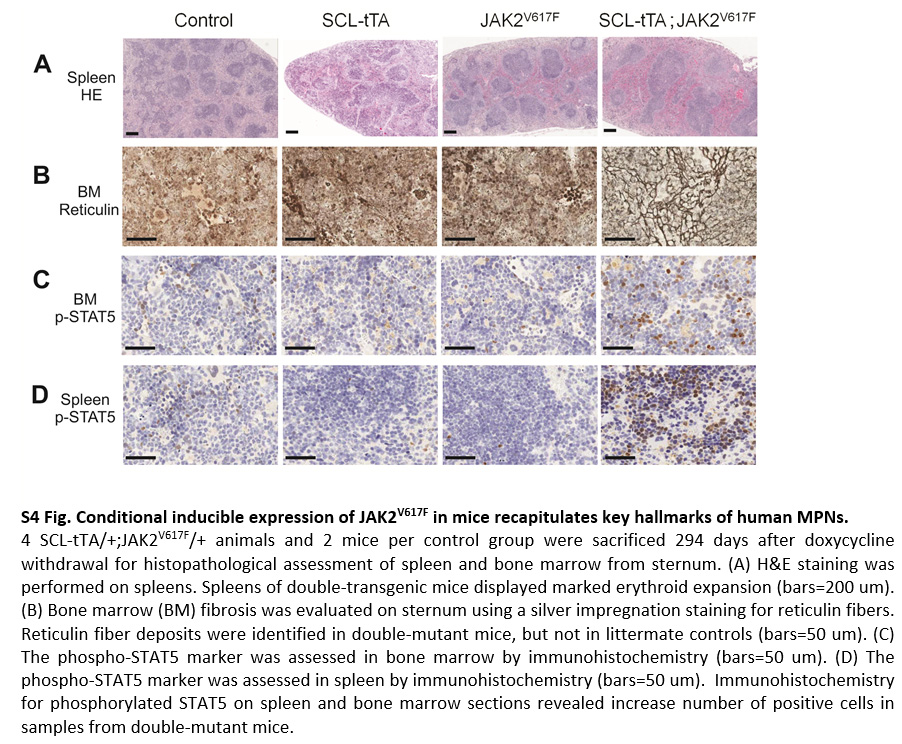

Supplement: S4 Fig — (JPG) [file pone.0221635.s004.jpg]

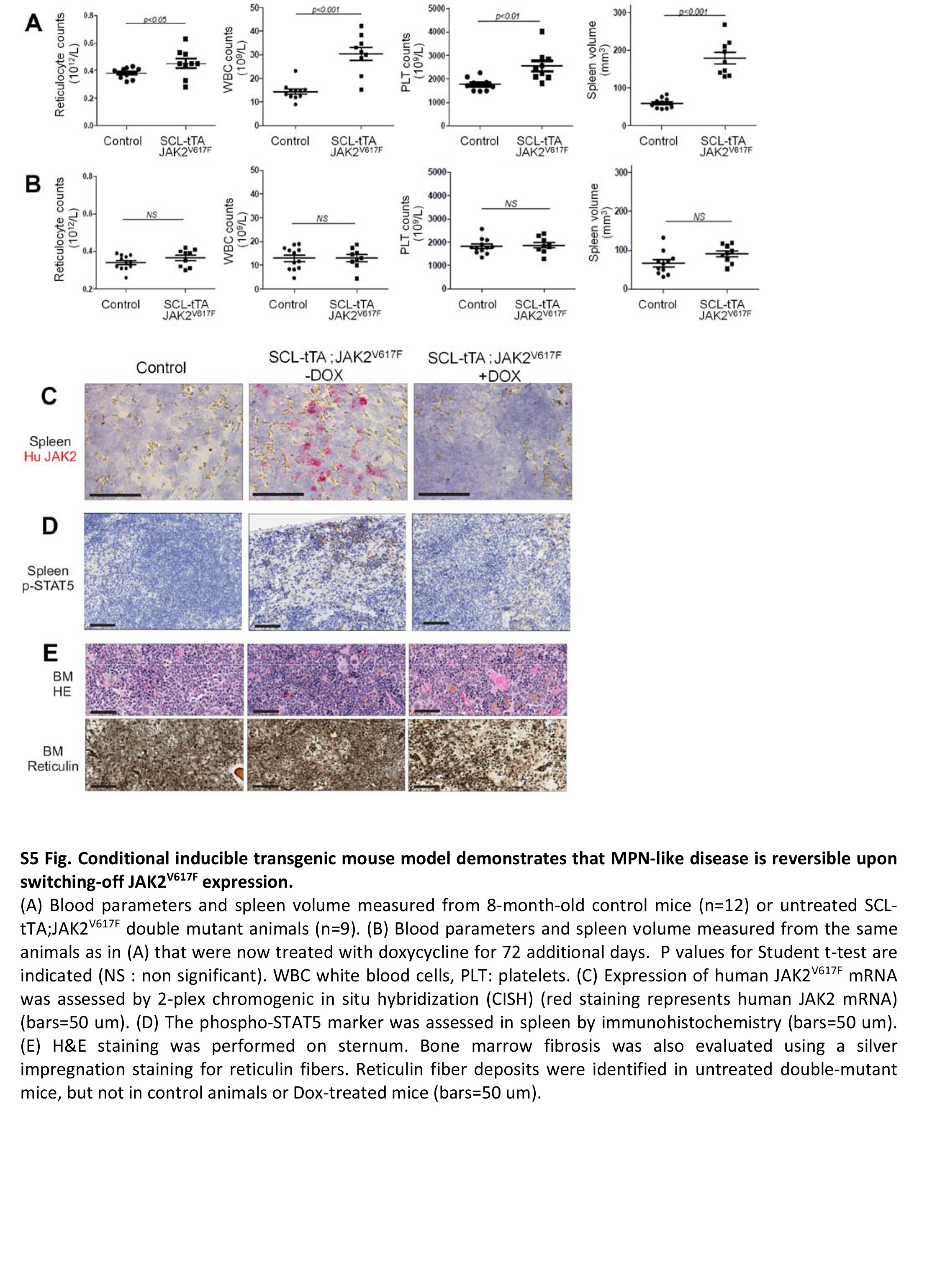

Supplement: S5 Fig — (JPG) [file pone.0221635.s005.jpg]

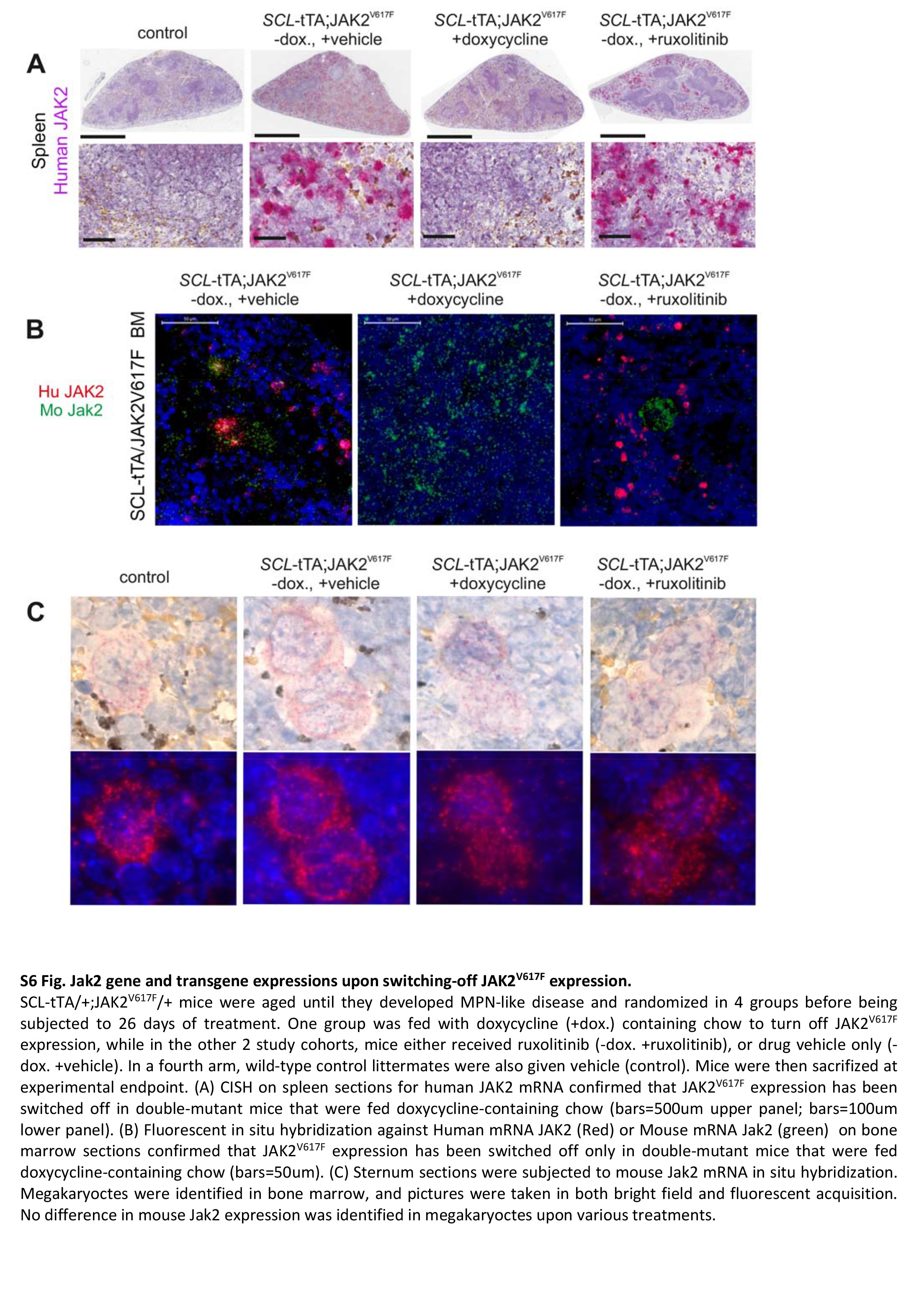

Supplement: S6 Fig — (JPG) [file pone.0221635.s006.jpg]

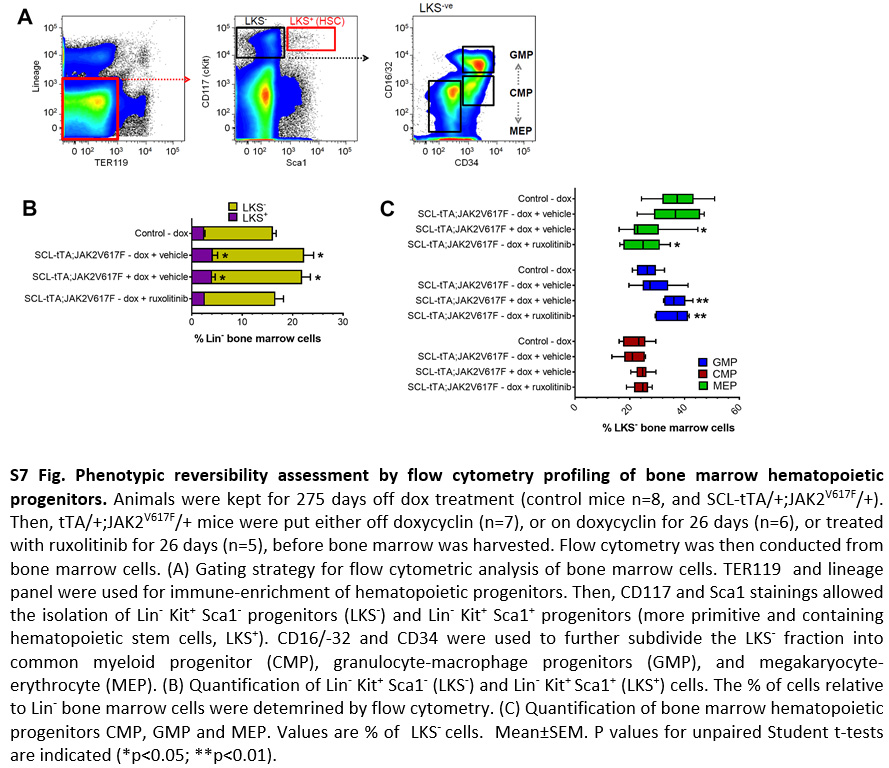

Supplement: S7 Fig — (JPG) [file pone.0221635.s007.jpg]

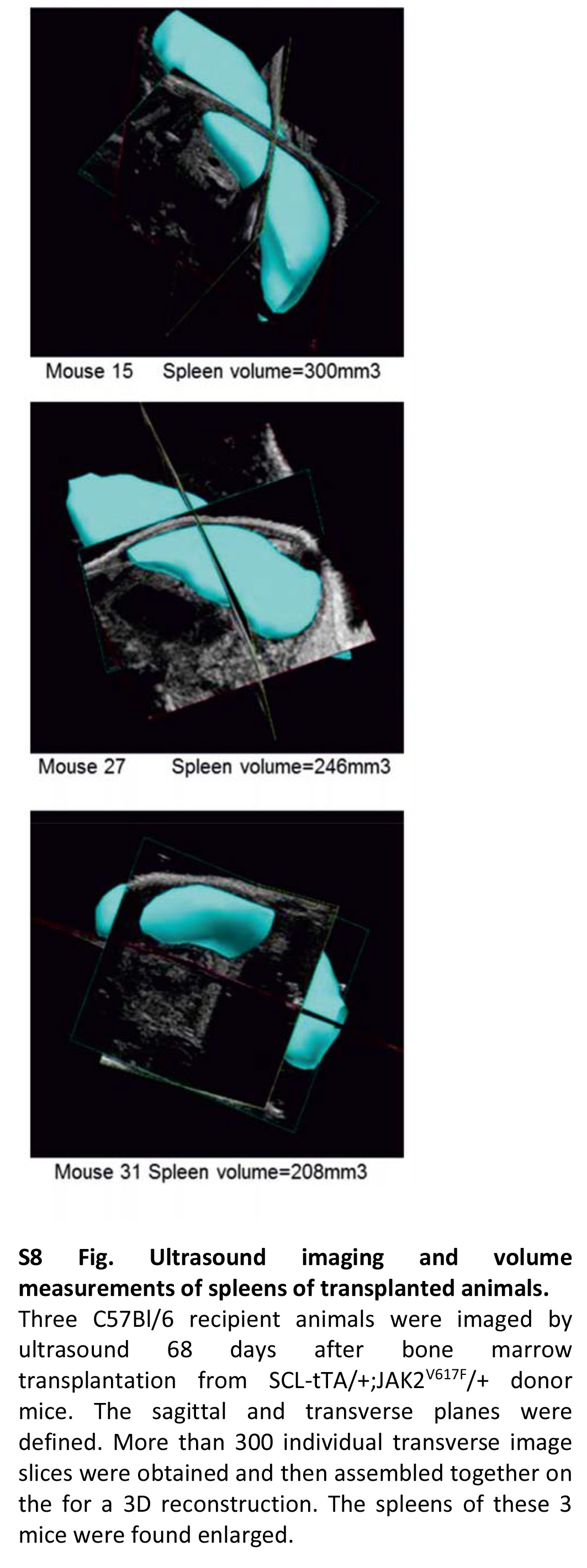

Supplement: S8 Fig — (JPG) [file pone.0221635.s008.jpg]

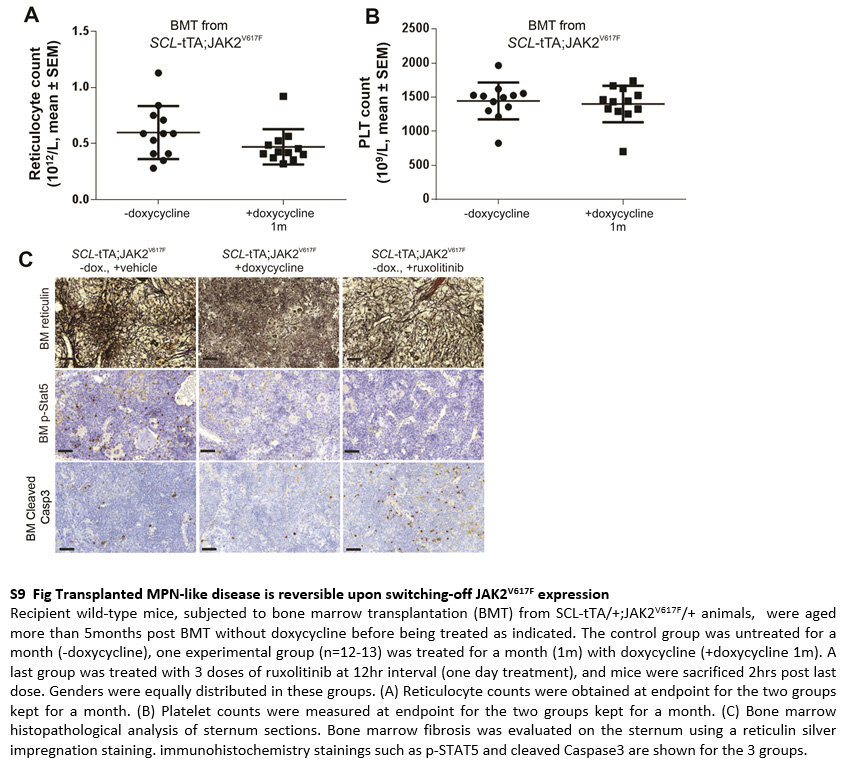

Supplement: S9 Fig — (JPG) [file pone.0221635.s009.jpg]

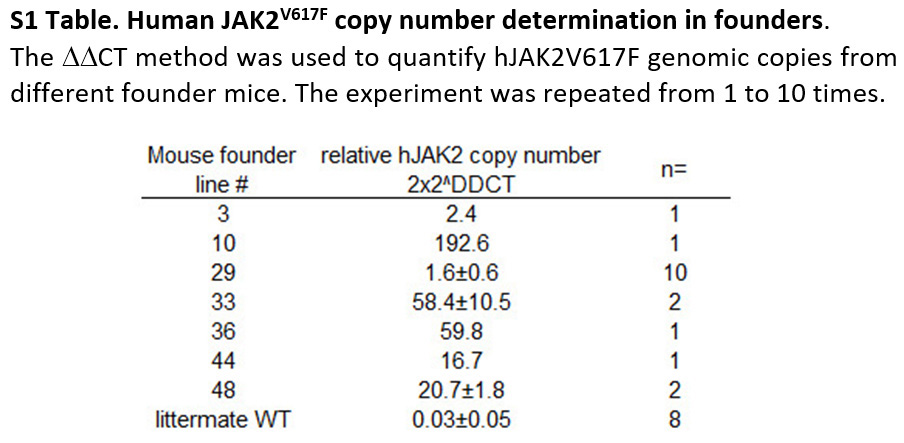

Supplement: S1 Table — (JPG) [file pone.0221635.s010.jpg]

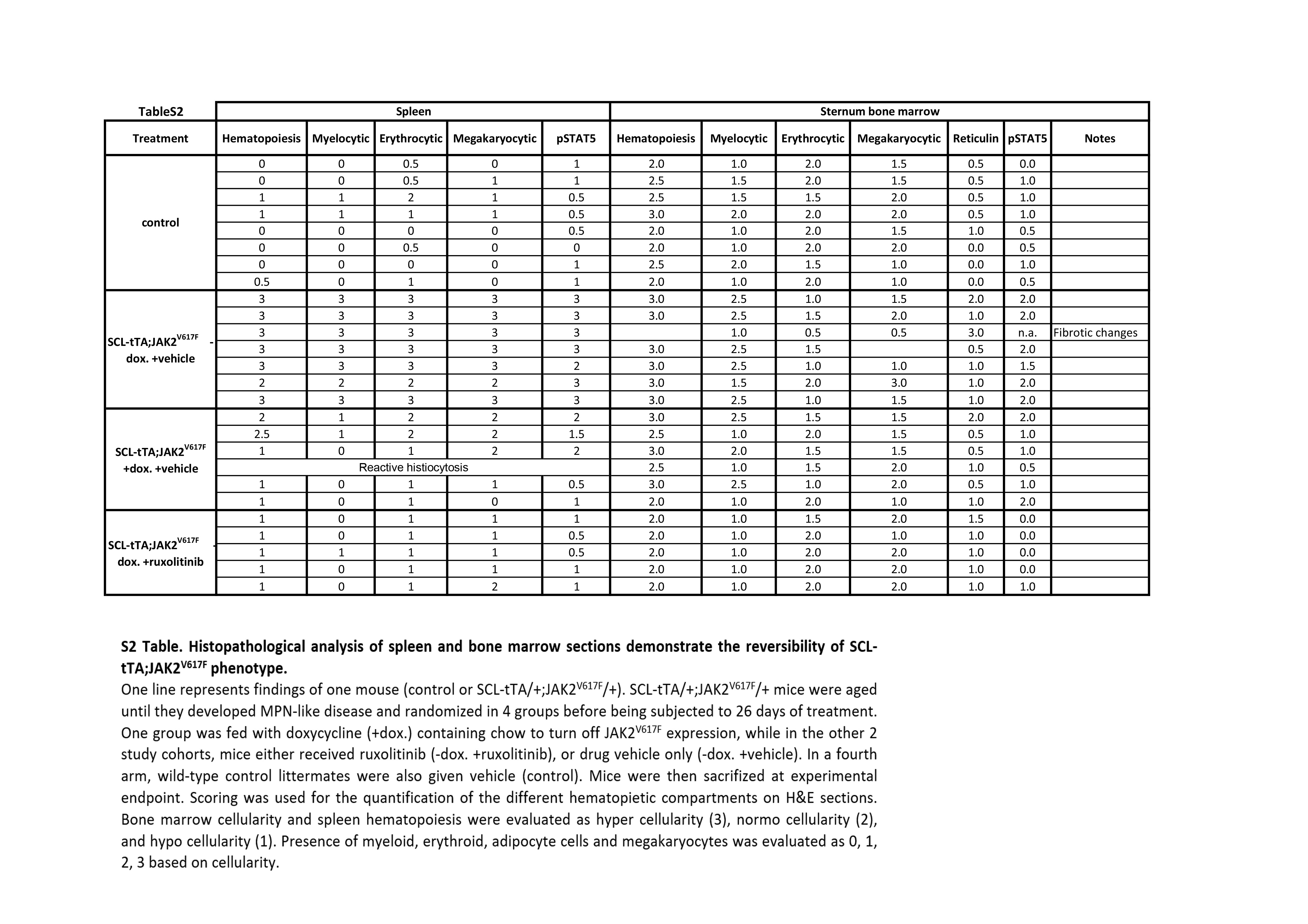

Supplement: S2 Table — (JPG) [file pone.0221635.s011.jpg]

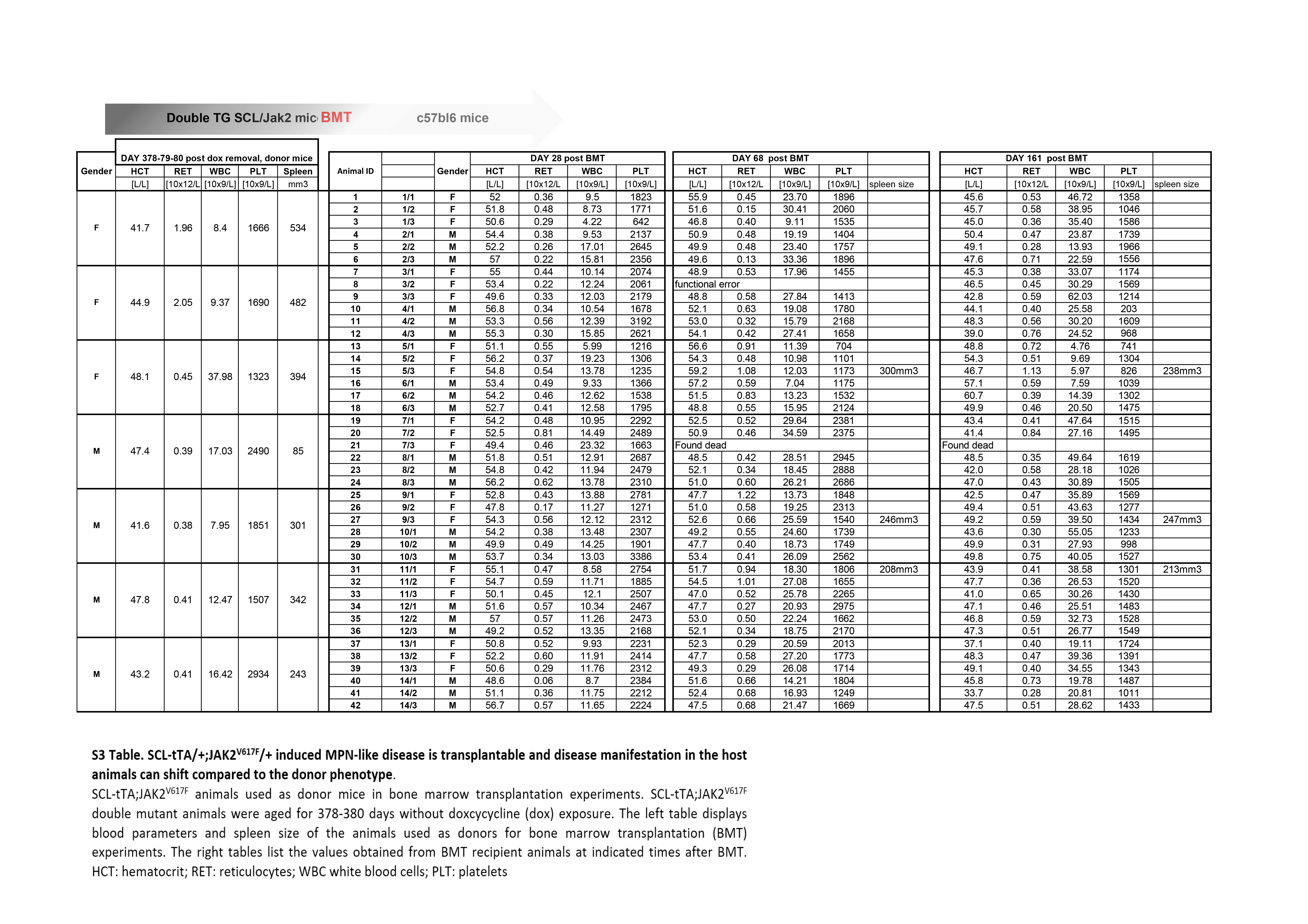

Supplement: S3 Table — S1 Methods (JPG) [file pone.0221635.s012.jpg]
