# Supplementary material for: A conditional inducible JAK2V617F transgenic mouse model reveals myeloproliferative disease that is reversible upon switching off transgene expression
Source: PLoS One. 2019 Oct 10;14(10):e0221635. doi: 10.1371/journal.pone.0221635 (PMC6786561; doi:10.1371/journal.pone.0221635)
Supplement: S1 Methods — (DOC) [file pone.0221635.s013.doc]

# S1 Methods

**Generation of the construct and reversible JAK2^V617F^ transgenic mice**

The human *JAK2*^V617F^ cDNA was inserted into pCe-lox511-CMVmin. An IRES-EGFP was added. The final plasmid pCe-lox511-CMVminTo-JAK2V617F-IRES-EGFP-FNeoF-loxP was digested with SbfI and BstBI enzymes before pronuclei microinjection. The microinjection was done in male C57Bl/6 pronuclei. Injected embryos were implanted into pseudopregnant females. 48 transgenic founders were obtained and presence of transgene was assessed by PCR. The founder line 29, B6-Tg(TetOCMV-JAK2V617F-IRES-EGFP)29Npa, was used for further experiments. For confirmation of integration of the full construct, mouse genomic DNA from progenies was used, and JAK2-GFP region corresponding to nucleotides 550-5307 of the construct pCe-lox511-CMVminTo-Jak2V617F-IRES-EGFP-FNeoF-loxP was amplified and sequenced. The sequence alignment revealed no differences compared to the reference sequence (data not shown).

**Breeding and generation of experimental mice**

SCL-tTA-2S knock-in was previously generated and characterized by Bockamp E et al [1]. and named Tal1^tm1(tTA)Bock^ [1]. B6.129S1-Tal1^tm1(tTA)Bock^ was backcrossed to C57Bl/6 background. The B6-Tg(TetOCMV-JAK2V617F-IRES-EGFP)29Npa and B6-Tal1^tm1(tTA)Bock^ mouse lines were then crossed and double heterozygous animals were generated as experimental animals.

**Human *JAK2* copy number determination by qPCR and** ΔΔ**CT method**

Quantitative real time PCR was performed on genomic DNA under standard PCR conditions with the real time PCR system. Relative human *JAK2* copy number in transgenic mice were determined using an adaptation of the ΔΔCT method for relative quantitation.

qPCR conditions:

0.8 ng of genomic DNA were amplified with forward and reverse primers at optimal concentrations (300 nM for *mJak2, hJAK2* and *mL1*), and diluted in ExpressSYBR-Green ER qPCR SuperMix Universal (Invitrogen) to 10 µl final reaction volume. All samples were run in triplicate under Two-Step PCR conditions (1 cycle 5 minutes at 95°C and 40 cycles: 10 seconds at 95°C and 30 seconds at 60°C) on Applied Biosystem 7900HT Fast Real-Time PCR System. After PCR each sample was analyzed by a dissociation protocol to check the specificity of the PCR product obtained. CT values (cycle threshold) were determined for each sample by the ABI 7900HT SDS software version 2.4.

List of primers used:

| **Name** | **Sequence** | |
| --- | --- | --- |
| human *JAK2* | Forward | GCCGAGTTGTAACTATCCATAAGCA |
|  | Reverse | TGACACGAAAGACAAAGCTTCCCT |
| mouse *Jak2* | Forward | TTCACGTTTCTTGTCTCCCTTTGCC |
|  | Reverse | ACCAAATGCTTGTGAGAAAGCTGAC |
| mouse *L1 repetitive element* | Forward | GTTACAGAGACGGAGTTTGGAG |
|  | Reverse | CGTTTGGATGCTGATTATGGG |

Adaptation of the ΔΔCT method for relative quantitation:

For each sample, the quantity (CT *mJAK2*) of the calibrator gene (mouse *JAK2*) was normalized against the quantity (CT *mL1*) of a reference gene (mouse *L1* repetitive element). ΔCT calibrator (CT *mJak2* - CT *mL1*) was obtained, as well as the quantity (CT *hJAK2*) of the target gene (human *JAK2*) and ΔCT sample (CT *hJAK2* - Ct *mL1*) was calculated. For each sample, the ratio between the quantity of human *JAK2* and the quantity of mouse *Jak2* was determined (2^-ΔΔCt^ method), as relative copy number CN = 2x2^-ΔΔCt^ .

**Primary mouse fibroblast culture assay to screen for transgene inducibility**

Ears of mice were swabed with 75 % EtOH and ear punch was collected. Tissues were immerse in 70 % EtOH for 2 minutes, then washed twice in PBS without Ca2+ or Mg2+. PBS was removed and tissues were cut into small pieces using a sterile scalpel into 10 cm cell culture dishes with 12 ml RPMI 1640 medium / 20% FCS / 3x Pen/Strep and 1 mg/ml collagenase typ Ia (Sigma) with 5 mM CaCl2, add 5 ug/ml fungizone. After overnight incubation (16-20hrs) at 37C, cells were disrupted by pipetting up and down and transfered in a tube with 3 ml RPMI medium. Cells were centrifuged at 1500 rpm for 5 minutes, washed and resuspended in 10 ml RPMI medium (with 20 % FCS, 3x Pen/Strep with fungizone) for plating. 30hrs later, adherent cells were washed with 1x PBS and fresh RMPI medium (with 20 % FCS, 3x Pen/Strep with fungizone) was added. Approximatively 2 weeks later 0.13 million cells were plated in wells of 6-well plate with medium without Pen/Strep and Fungizone for transfection. The next day Lipofectamine 2000 transfection was perfomed with 2 ug pcDNA3.1/Hygro itTA. 30hrs later, cells were harvested used for genotyping, western-blotting and RT-qPCR.

**Gene expression evaluation by RT-qPCR**

RNA extraction was done with the PicoPure RNA Isolation kit (ThermoFisher #KIT0204). RNA samples were reverse-transcribed using Sensiscript RT kit (Qiagen #205213). Real time PCR for detecting mRNA levels was performed on an AB Applied Biosystems 7900 HT cycler. Primers are listed below. Each sample was run in triplicate. Difference in cDNA input was compensated by normalizing to the expression of mouse HPRT gene, or mouse Jak2 as indicated.

hJAK2-qPCR-F GTGGCAGCAACAGAGCCTATC

hJAK2-qPCR-R GGAGCTTCAGCACCTCGAGAT

mJAK2-qPCR-F TGGCAGCAGCAGAACCTACA

mJAK2-qPCR-R GGAGCTTCAGCCCCACG

EGFP-qPCR-F CTGCTGCCCGACAACCA

EGFP-qPCR-R TGTGATCGCGCTTCTCGTT

Murine HPRT-F GCTCGAGATGTCATGAAGGAGAT

Murine HPRT-R AGCAGGTCAGCAAAGAACTTATAGC

**Extraction of protein lysates, and Western blotting**

Cells or snap-frozen samples were homogenized in RIPA buffer supplemented with 2 mM sodium vanadate, 0.1 mM PMSF and one protease inhibitor tablet per 10 ml (Roche catalogue number 11836 145 001). Total protein content of the homogenates was measured using BCA protein assay kit (Novagen catalogue number 71285-3). For For western blotting, 30 or 40 µg protein extracts were used for immunoblotting analysis. The list of antibodies used is as follow: rabbit anti-JAK2 (Cell Signaling Technologies Inc., Danvers, MA, #3230), mouse anti-GFP (Roche #11814460001), mouse anti β-tubulin (# T4026) from Sigma (St. Louis, MO, USA), tTA was recognized by TetR Monoclonal Antibody (Clone 9G9, Takara). Secondary antibodies from Amersham: anti-mouse NA931V and anti-rabbit NA934V were used as well as ECL reagents from Amersham RPN2106.

**Experimental animal studies**

All animal studies were conducted in accordance to procedures covered by permit number BS-2139 and BS-2287 issued by the Kantonales Veterinäramt Basel-Stadt and strictly adhered to the Eidgenössisches Tierschutzgesetz and the Eidgenössische Tierschutzverordnung. All animals had access to food and water ad libitum and were identified with transponders. Mice were housed in a specific pathogen–free facility with a 12-h light/12-h dark cycle. Animals were monitored carefully and regularly in all experiments.

**Genotyping of animals**

DNA was extracted from tail clips and was digested with proteinase K. SCL-tTA-S2 was detected by PCR (annealing temperature of 58C) with the following primers 5'-ccctgctcgatgccctggc-3' and 5'-aggaaggcaggttcggctcc-3'. An amplicon of 420bp was generated in presence of the knock-in allele. Human JAK2 was genotyped by PCR (annealing temperature of 58C) with the following primers 5'-ACTAAATGCTGTCCCCCAAAG-3' and 5'-CCCATGCCAACTGTTTAGC-3'. An amplicon of 480bp was generated in presence of the transgene.

**Ultrasound imaging of the spleen in vivo and measurement of spleen dimensions**

Mice spleens were imaged by ultrasound using a Vevo2100® High‐Resolution Imaging System (VisualSonics, Toronto, Canada) following the manufacturer's instructions. Briefly, mice were starved 3 hours before imaging and all fur was removed from the abdomen of mice using depilatory cream (Nair), then the skin was washed with water to remove excess lotion. Mice were maintained anesthetized for the duration of the imaging (1.5% isoflurane ; 0.8L O2) on the heated imaging platform to preserve body temperature. With a generous amount of warmed ultrasound gel applied to the skin, the mice were restrained with surgical tape and ultrasound images were taken of its abdomen using the MS500D transducer equipped with 3D Motor. The sagittal and transverse planes are defined. More than 300 individual transverse image slices are obtained and then assembled together on the VevoLAB software for a 3D reconstruction.

**Animal treatments**

The expression of the transgene was switched off by doxycycline treatment. Mice were given doxycycline food pellet from Provimi Kliba. The food contained doxycycline at a concentration of 200mg/kg. Ruxolitinib was administrated at 60mg/kg in 0.5% hydroxypropyl methyl cellulose (HPMC) orally twice a day in a solution of 10ml/kg. SCL-tTA;JAK2^V617F^ double mutant animals presenting MPD-like phenotype (splenomegaly and significant changes in blood counts) upon withdrawal of doxycycline were used in ruxolitinib efficacy studies. Animals were randomized based on the hematocrit readout. Response was reported with the measure of blood parameters or spleen volume using ultrasound imaging. On the bone marrow transplantation models, dosing of transplanted mice started 21 to 28 days following transplant when disease was established by blood count.

**Bone marrow transplantation**

Bone marrow cells were isolated from tibias and femurs of 15-17-month-old SCL-tTA / JAK2^V617F^ mice under sterile conditions. The bones were crushed in a mortar with RPMI + 10% FCS. The medium containing bone marrow cells was filtered through a cell strainer (BD Falcon, # 352340). Cells were centrifuged, the supernatant was removed, and red blood cell lysis buffer was added for 10 minutes at room temperature (Stemcells technologies #07800). Then, RPMI + 10% FCS was added and cells were washed and centrifuged again. Cells were resuspend in pure sterile PBS, counted using Sysmex Nucleocounter, and aliquoted for bone marrow transplantation.

8-9 weeks old C57Bl/6 recipient mice were subjected to total body irradiation (Biobeam, GSM Gamma-Service Medical GmbH) on the day of cells transplantation (2 doses of 4,5Gy at 4hrs interval). Immediately following the second irradiation, mice received a transplant of 5x10^6^ bone marrow cells (from aged SCL-tTA / JAK2^V617F^ double mutant donor animals), injected in tail vein i.v. in a total volume of 100ul in HBSS (Gibco®, Cat#14175) at pH 7.4. Body weights were monitored daily for 2 weeks post-transplant. Mice were given antibiotics (Bactrim (Roche Pharma AG) 5 ml in 250 mL drinking water) to prevent infections for 14 days post bone marrow transplant, and DietGel Boost.

**Blood counts**

Blood was collected from the tail vein using Microvette 100K3E tubes with EDTA (SARSTEDT, cat#. 20.1278) for blood analysis. Blood parameters (red blood cells, white blood cells, platelets, and reticuocytes counts) were determined on Sysmex blood analyzer.

## Histopathological assessment

Sterna, femurs and spleens were collected, fixed in formalin (10 % neutral buffered formalin (NBF)) for 48 hours at room temperature, EDTA decalcified for 3x24 hours at 37°C for bones, rinsed in PBS, processed for dehydration, cleared and paraffinized.

After embedding in paraffin, 3µm sections were prepared. H&E staining was performed on all organs. For histological evaluation, bone marrow cellularity was evaluated as hyper cellularity (3+), norm cellularity (2+), and hypo cellularity (1+). Presence of myeloid, erythroid, adipocyte cells and megakaryocytes was evaluated as 0, 1+, 2+, 3+ based on cellularity. Bone marrow fibrosis was evaluated on the sternum using a silver impregnation kit for reticulin fibers (Bio-Optica, #04-04080).

## Immunohistochemistry

# 3um thickness tissue section were stained on a BondRX platform (Leica Biosystems) using Epitope Retrieval 2 (ER2) conditions for 20mn at 100°C for all tested markers. The detection system used was either Refine DAB kit (Leica Biosystems) for p-STAT5 and Cleaved Caspase 3 immunohistochemistry or Refine Red kit (Leica Biosystems) for GFP immunohistochemistry. Slides were hematoxylin counterstained, dehydrated and cover slipped. Primary antibodies used were anti-phospho-STAT5 (clone C11C5, CST, #9359) or cleaved Caspase3 (CST, #9661) or GFP (MBL, #598).

**mRNA In Situ Hybridization**

In situ hybridization was performed using either the 1-plex Quantigene View RNA ISH Tissue assay kit (Affymetrix, #QVT0051) or the 2-plex Quantigene View RNA ISH Tissue Assay kit (Affymetrix, #QVT0012). Assays were performed as per the manufacturer’s instructions with stock solutions. Before beginning the hybridization protocol, 5um thickness tissue section were baked at 60°C for 30 min to increase tissue adhesion. The paraffin was removed from the slides with xylene before being boiled in a pretreatment solution (Affymetrix) for 10 min and incubated with Protein Kinase K (Affymetrix) at 40°C for 10 min. Custom probes for Human JAK2 and Mouse Jak2 (Affymetrix) were then hybridized to the tissue. Signal amplification was accomplished by hybridizing Type1 and Type2 specific pre-amp oligonucleotides, amp oligonucleotides and labeled oligonucleotides sequentially. Slides were then mounted with an aqueous mounting medium (Dako, Ultra mount). Digital slide image data was generated from the glass slides using the Aperio AT2 slide scanner (Leica Biosystems) for bright field, and the P250 Scanner (3D Histech) for fluorescent acquisition.

**Flow cytometry analysis and sorting.**

Mouse tibiae were used to extract cells for flow cytometry. Multicolour analysis for progenitor and stem-cell quantification was performed on a FACS-Aria (BD Biosciences, San Jose, CA). Cells were stained in PBS 2%BSA with CD34–FITC (1:100), CD150–PE (1:100), FcγRIII/II-PercpCy5.5 (1:400), CD135-PECY5 (1:100), CD117-PECy7 (1:200), TER119-APCCy7 (1:500), lineage cocktail-APC (B220/CD19/CD3/CD4/CD8/NK1.1/TER119/CD11b/Gr1), Sca1-biotin (1:500), streptavidin-Brilliant Violet 605 (1:500) and 1 μg ml-1 DAPI. FACS enrichment of LKS, GMP, CMP and MEP was performed on the FACS ARIA and data analyzed using FlowJo software (Tree star, Ashland, OR). Briefly, bone marrow (depleted of red blood cells) was labeled with APC-conjugated mature blood cell lineage-specific antigens CD3, CD4, CD8, Gr-1, Mac-1, B220, CD19 and TER119 . Doublets, DAPI positive dead cells, and cells expressing lineage markers were excluded from the analysis.

| *Antigen* | *Conjugate* | *Clone* | *Supplier* | *Specificity* |
| --- | --- | --- | --- | --- |
| CD41 | FITC | MWReg30 | BD | Megakaryoctes  Platelets |
| CD34 | FITC |  | BD | HSC |
| F4/80 | PerCP/Cy5.5 | BM8 | Biolegend | Pan macrophage marker |
| CD16/32 | PerCP/Cy5.5 | 93 | Biolegend | FcRIII  (B, Mono; Mac, NK, Gran, Mast & DCs) |
| CD71 | PE | C2F2 | BD | Erythroid precursors |
| CD150 | PE | TC15-12F12.2 | Biolegend | SLAM |
| CD135 | PE/Cy5 |  | Ebioscience | Flt3R/Klk2 |
| Ly6G | PE/Cy7 | 1A8 | Biolegend |  |
| CD117 | PE/Cy7 |  | Ebioscience | ckit |
| CD3 | APC | 145-2C11 | Biolegend | T cell lineage |
| CD4 | APC | GK1.5 | Biolegend | T cell lineage |
| CD8 | APC | 53-6.7 | Biolegend | T cell lineage |
| Gr1 | APC | RB6-8C5 | Biolegend | Ly6G |
| CD11b | APC | M170 | Biolegend | aMb2 |
| B220 | APC | RA3-6B2 | Biolegend | CD45R |
| CD19 | APC | ID3 | Biolegend | B cells |
| TER119 | APC | TER119 | Biolegend | Erythrocytes |
| TER119 | APC/Cy7 | TER119 | Biolegend | Erythrocytes |
| CD19 | biotin | ID3 | Biolegend | B cells |
| Sca1 | biotin | D7 | Biolegend | Ly6A/E |
| Streptavidin | Brilliant Violet 605 |  | Biolegend | Biotin |

**Hematopoietic Cell Colony-Forming Assays**

Mouse tibiae were used to extract bone marrow cells and phenotypically defined hematopoietic progenitor-cell populations were obtained by FACS. Myeloid lineage colony-forming assays were performed in SCF, IL-3, IL-6, EPO supplemented methylcellulose-based medium (Methocult M3434, Stem Cell Technologies). Doxycycline (1ug/ml) and ruxolitinib (1μmol/l) were applied to the medium where indicated and replaced every three days. For LKS-, CMP, GMP and MEP methylcellulose cultures, 2000 cells were seeded in duplicate and scored for colony formation at 7–10 days. For serial replating experiments, LKS+ cells were plated at 2,000 cells per well and scored for colony formation after 10-12 days of primary culture. Cells were subsequently counted and resuspended in fresh medium with 2 x 10^5^ cells replated for each of three rounds of serial replating. Serial plating efficiency was scored as percentage of initial CFU per well obtained at 10-12 days culture.

**Reference**

1. Bockamp E, Antunes C, Maringer M, Heck R, Presser K, Beilke S, et al. Tetracycline-controlled transgenic targeting from the SCL locus directs conditional expression to erythrocytes, megakaryocytes, granulocytes, and c-kit-expressing lineage-negative hematopoietic cells. Blood. 2006;108(5):1533-41.
